# All‐Optical Control of Bidirectional Polarization Switching in Ferroelectric Heterostructures for Neuromorphic and In‐Memory Computing

**DOI:** 10.1002/advs.202522092

**Published:** 2026-01-29

**Authors:** Jingjie Niu, Jiahui Lyu, Jie Li, Koyal Suman Samantaray, Cheolhwa Jang, Yoonmyung Lee, Ji‐Sang Park, Sungjoo Lee

**Affiliations:** ^1^ SKKU Advanced Institute of Nanotechnology (SAINT) Sungkyunkwan University Suwon South Korea; ^2^ Department of Nano Science and Technology Sungkyunkwan University Suwon South Korea; ^3^ IMEC Heverlee Belgium; ^4^ Department of Electrical and Computer Engineering Sungkyunkwan University Suwon South Korea; ^5^ Department of Nano Engineering Sungkyunkwan University Suwon South Korea

**Keywords:** all‐optical bidirectional polarization switching, artificial visual system, logic‐in‐memory, neuromorphic computing, van der Waals heterostructure

## Abstract

All‐optical in‐memory computing is emerging as a critical technology for next‐generation energy‐efficient and high‐speed information processing because it avoids frequent optical‐electrical‐optical conversions and integrates sensing, processing, and memory within a single device. Here, we report the demonstration of bidirectional polarization switching in a van der Waals heterostructure composed of ferroelectric CuInP_2_S_6_ (CIPS) and semiconducting MoS_2_. Wavelength‐tunable excitation (660–405 nm) enables robust, bidirectional polarization reversal through the interaction between the photogenerated charges in MoS_2_/CIPS heterostructure and ferroelectric polarization charges in CIPS. Two wavelength‐dependent carrier dynamic mechanisms were established specifically for excitations below and above the CIPS bandgap. These mechanisms result in opposite charge accumulation at the interface, leading to opposite polarization switching directions. The device demonstrates high‐performance all‐optical nonvolatile memory. Furthermore, it emulates all‐optical controlled retina‐like synaptic plasticity, including paired‐pulse facilitation/inhibition, short‐term and long‐term potentiation and depression, and learning‐forgetting behaviours, with wavelength‐selective long‐term potentiation and depression enabling neuromorphic image recognition. Additionally, the single device implements reconfigurable all‐optical controlled Boolean logic gates.

## Graphical abstract

We propose an optical ferroelectric field‐effect transistor, composed of a MoS_2_/CIPS heterostructure, demonstrates the feasibility of all‐optical nonvolatile memory, neuromorphic computing, and logic‐in‐memory operations. The device exhibits reversible light‐controlled memory states, retina‐like synaptic plasticity, and wavelength‐selective potentiation/depression for neuromorphic image recognition. Furthermore, purely optically controlled, reconfigurable Boolean logic gates with in situ memory were realized within a single device.

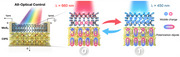

## 1 Introduction

The rapid development of artificial intelligence and the Internet of Things has created an urgent demand for next generation computing architectures that can overcome the inherent limitations of conventional von Neumann systems. Light‐controlled memory and in‐memory computing devices have attracted significant attention due to their ability to directly integrate sensing, processing, and memory within a single platform. By utilizing light as the information carrier, these devices enable ultrafast signal processing, high‐bandwidth communication, and energy‐efficient parallel computation, offering unique opportunities to realize both neuromorphic functionalities and logic‐in‐memory operations within photonic hardware [1, 2, 3, 4, 5].

Although light‐stimulated memory devices have been extensively studied, most reported systems rely on hybrid photonic‐electric control schemes, where light is used to induce potentiation (long‐term enhancement of synaptic weight) and electrical bias is applied to induce depression (weight reduction) [4, 6, 7, 8, 9, 10]. While effective, these hybrid strategies inevitably suffer from optical‐electrical‐optical conversions, which limit overall operation speed, increase energy consumption, and complicate device integration [11, 12, 13, 14]. To fully exploit the potential of photonic computing, it is highly desirable to develop fully light‐controlled memory and in‐memory computing systems, in which both potentiation and depression, as well as logic‐in‐memory and logic state reconfiguration, are achieved exclusively through optical stimuli.

Recent efforts toward all‐optical neuromorphic devices have focused on exploring various material platforms and mechanisms. For example, all‐optically driven neuromorphic computation has been demonstrated in black phosphorus through the formation of oxidation‐induced defects and charge‐trap states [3]. A bidirectional synaptic phototransistor based on the 2D ferroelectric semiconductor α‐In_2_Se_3_ has also been developed, utilizing interlocked in‐plane and out‐of‐plane polarizations to enable gate‐tunable barrier heights [14]. In addition, heterostructures composed of organic polymers or inorganic oxide semiconductors have been employed to achieve all‐optical plasticity via energy band alignment and carrier transfer, light‐induced interfacial carrier trapping, or light‐induced defect‐state modulation [1, 5, 11, 12, 13, 15, 16, 17, 18, 19, 20, 21, 22, 23, 24, 25, 26, 27, 28]. Despite these advances, several challenges remain: BP suffers from environmental instability and limited device performance; *α*‐In_2_Se_3_ phototransistors still require electrical assistance; and semiconductor heterostructure‐based optical synapses often exhibit volatile responses and poor nonlinearity control due to metastable trap states [14]. These limitations significantly hinder scalability and practical integration into large‐scale neuromorphic hardware.

Ferroelectric materials provide a promising pathway to overcome these limitations due to their intrinsic nonvolatile and switchable polarization states. However, reported works on optical manipulation of ferroelectric polarization have so far demonstrated only unidirectional polarization control under optical stimulation [7, 29, 30, 31, 32, 33]. Consequently, in optical neuromorphic devices based on ferroelectrics, optical pulse excitation could merely induce potentiated synaptic weight updates, whereas light‐controlled depression remained unachieved.

Here, we report all‐optical controlled bidirectional polarization switching in a van der Waals (vdW) heterostructure composed of ferroelectric CuInP_2_S_6_ (CIPS) and semiconducting MoS_2_. Benefiting from the suitable bandgaps of CIPS (≈2.8 eV) [34] and MoS_2_ (≈1.2 eV) [35], the heterostructure exhibits photoresponse in the visible and near‐UV spectral ranges. Robust bidirectional polarization switching was achieved under wavelength‐tunable excitation from 660 to 405 nm, as directly confirmed by contact resonance piezoresponse force microscopy (PFM). The underlying mechanism is attributed to wavelength‐dependent polarization switching based on the interaction between the photogenerated charges in the MoS_2_/CIPS heterostructure and ferroelectric polarization charges in CIPS. Two wavelength‐dependent carrier dynamic mechanisms were established specifically for excitations below and above the CIPS bandgap. These two processes produce opposite charge accumulation at the MoS_2_/CIPS interface and drive polarization switching in opposite directions, thereby enabling robust and reversible all‐optical control of the ferroelectric polarization and channel conductivity.

The fabricated device demonstrated high‐performance all‐optical nonvolatile memory with an on/off ratio greater than 10, a retention time exceeding 10^4^ s, endurance over 10^4^ cycles, and multilevel storage capability achieved via light‐intensity modulation. In addition, the device emulated retina‐like synaptic plasticity through all‐optical analog modulation of ferroelectric polarization, successfully mimicking versatile synaptic functions such as paired‐pulse facilitation/inhibition (PPF/PPD), short‐term and long‐term potentiation and depression (STP/LTP, STD/LTD), and learning‐forgetting behaviors. Notably, the device exhibited wavelength‐selective LTP and LTD, enabling both image pre‐processing and neuromorphic image recognition. Moreover, purely optically reconfigurable Boolean logic gates (AND, OR, NAND, NOR) were demonstrated within a single device, highlighting its multifunctional capability for integrated photonic in‐memory computing.

## 2 Results and Discussion

### 2.1 All‐Optical Bidirectional Polarization Switching and Underlying Mechanism

**FIGURE 1:**
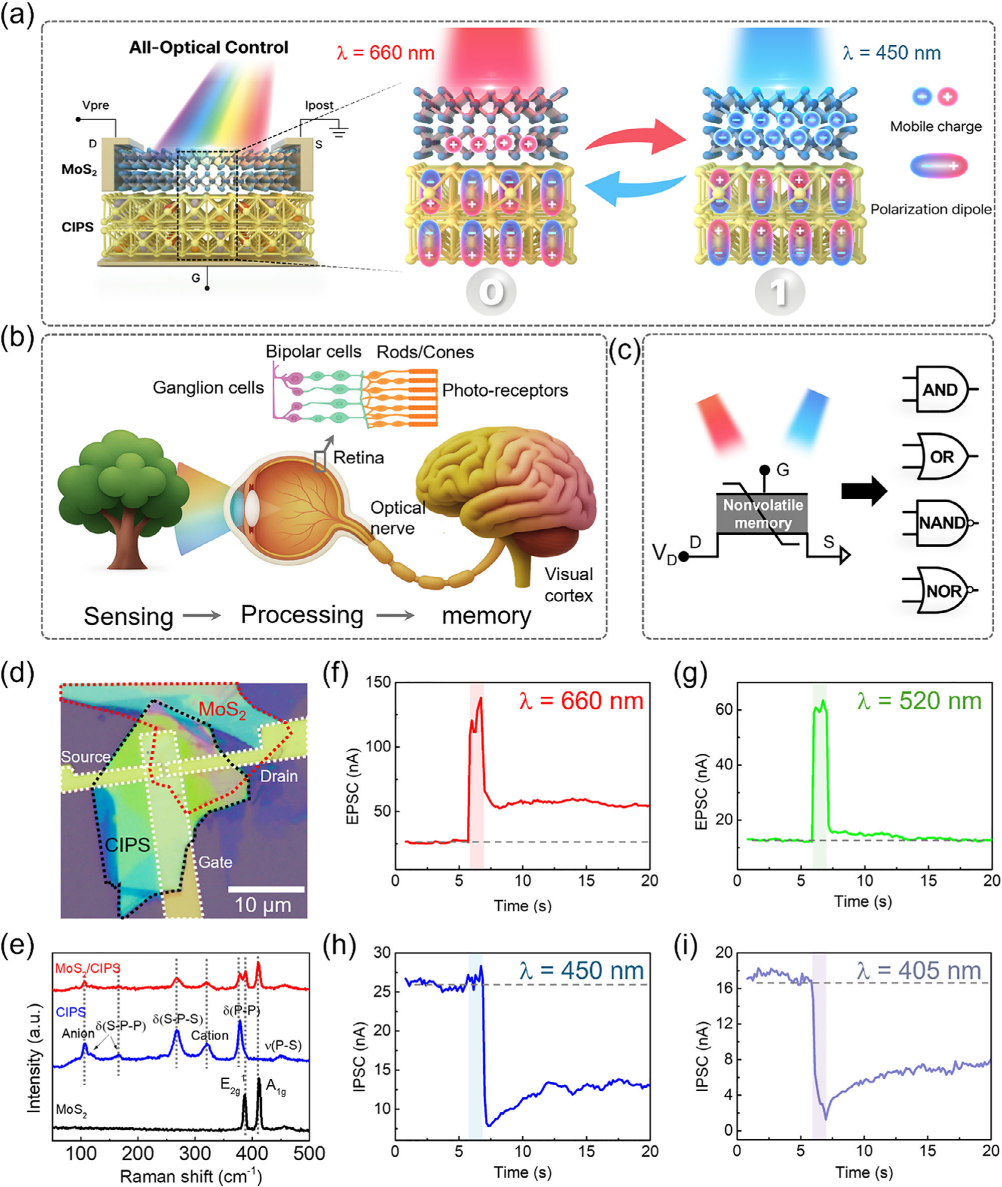
Working principle and fundamental characterization. (a) Left: structure of bottom‐gated vdW optical‐FeFET consisting of a semiconducting MoS_2_ and ferroelectric dielectric CIPS heterostructure. Right: all‐optical control of bidirectional polarization switching and high‐ and low‐resistance states. (b) Demonstration of an artificial visual system integrating sensing, processing, and memory in a single device. (c) All‐optical logic‐in‐memory operations. (d) Optical microscope image of a typical optical‐FeFET, with colored dotted lines highlighting the different components of the device. (e) Raman spectrum of MoS_2_, CIPS, and MoS_2_/CIPS heterostructure. (f)–(i) Postsynaptic current (PSC) responses of the device under optical stimulation at wavelengths of 660, 520, 450, and 405 nm, with a pulse duration of 1 s.

Figure 1a–c illustrates the working principle and in‐memory computing functionality of the vdW optical ferroelectric field‐effect transistor (optical‐FeFET) under wavelength‐dependent optical stimulation. The left panel of Figure 1a depicts the vdW heterostructure composed of semiconducting MoS_2_ and ferroelectric CIPS, which serves as the active platform for optical modulation. The right panel shows the polarization switching process in CIPS, where incident photons of different energies induce polarization switching in opposite directions. Polarization switching in our device is fully light‐driven, without any electrical bias, minimizing charge‐trapping, imprint, and fatigue effects associated with electrical stress while enabling highly reversible operation. In contrast, previously reported photo‐electrical hybrid schemes in ferroelectric device (Table S2) require an external voltage for reverse conductance changing [14] and typically allow only unidirectional or partial optical control [9, 29, 31, 33, 36]. In contrast, our device achieves fully optical reversible, wavelength‐selective, all‐optical polarization switching with nonvolatile retention—functionality that has not been demonstrated in prior works. Based on this unique behavior, the device demonstrates the feasibility of all‐optical nonvolatile memory (Figure 1a), an artificial visual system based on retina‐inspired optoelectronic neuromorphic computing (Figure 1b), and all‐optical logic‐in‐memory operations (Figure 1c). Specifically, the opposite directions of polarization switching enable the device to switch between high‐ and low‐resistance states. Beyond exhibiting wavelength‐selective synaptic plasticity, the device also emulates key processes of biological vision, thereby integrating sensing, processing, and memory within a single platform. Moreover, it realizes purely light‐controlled logic‐in‐memory operations, supporting the implementation of AND, OR, NAND, and NOR Boolean logic gates within the same architecture. One wavelength serves as the logic input, while the other functions as a pre‐programming signal; by alternating the pre‐programming signal, distinct Boolean logic functions can be implemented with in situ memory in a single device. This universal optical programmability highlights the potential of the device as a reconfigurable optoelectronic computing platform.

The optical microscopy image of the fabricated MoS_2_/CIPS heterostructured vdW FeFET is shown in Figure 1d, with colored dotted lines highlighting the device components. Atomic force microscopy (AFM) characterization of the vdW FeFET, presented in Figure S1, determined the thicknesses of MoS_2_ and CIPS flakes to be approximately 5 and 79 nm, respectively. The Raman spectra of mechanically exfoliated vdW MoS_2_ and CIPS flakes, as well as their heterostructures, are presented in Figure 1e. No noticeable Raman shift was observed in the heterostructure, indicating a high‐quality interface between the materials. Figure 1f–i present the postsynaptic current (PSC) responses of the device under optical stimulation at wavelengths of 660, 520, 450, and 405 nm, with a pulse duration of 1 s. The device exhibits both excitatory postsynaptic current (EPSC) and inhibitory postsynaptic current (IPSC) behaviors depending on the incident wavelength. By comparing the initial and final conductance states, light‐induced synaptic potentiation and inhibition can be clearly identified. Notably, a transition from potentiation to inhibition occurs with decreasing wavelength, whereas 660 and 520 nm illumination induce potentiation, 450 and 405 nm illumination result in inhibition. The PSC responses of the device under ten consecutive optical pulse stimulations at four different wavelengths are presented in Figure S2, further confirming the reproducibility and stability of the wavelength‐dependent potentiation and inhibition behaviors. Among these, the 660 and 450 nm light sources were employed as the primary control sources in the experiments, owing to their suitable modulation strength and superior linearity of PSC under consecutive optical pulse stimulations.

**FIGURE 2:**
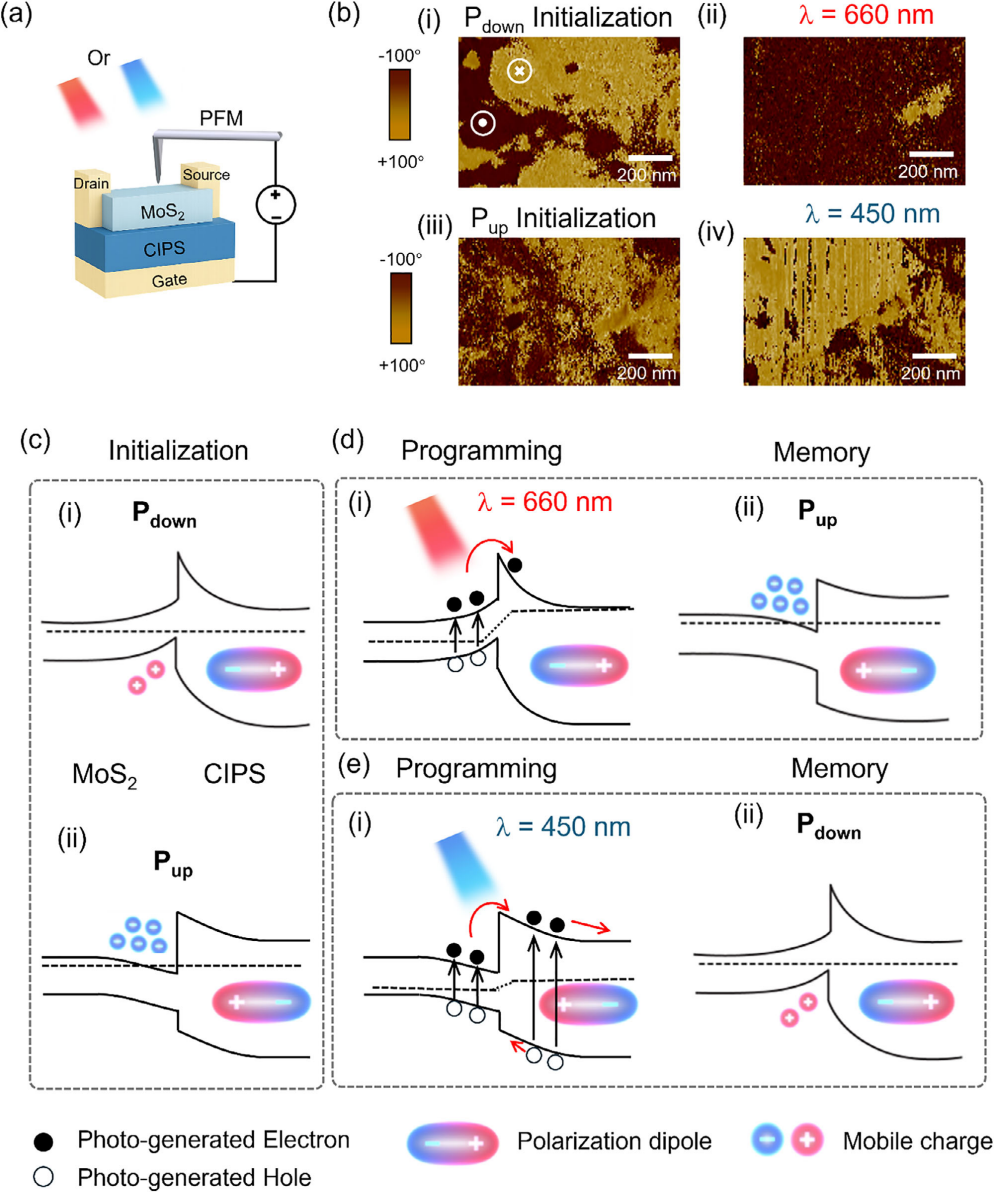
Mechanism of all‐optical bidirectional polarization switching. (a) Schematic of PFM measurement with in situ light exposure. (b) PFM phase scanning of the optical‐FeFET channel area with MoS_2_/CIPS heterostructure in dark ((i) and (iii)) or after light illumination with wavelength of 660 nm (ii) or 450 nm (iv). Illustration of the electric fields in the optical‐FeFET with MoS_2_/CIPS heterostructure and the corresponding band diagrams (c) under the dark condition (initialization), (d) longer‐wavelength lights (e.g., 660 nm) exposure, and (e) shorter‐wavelength lights (e.g., 450 nm) exposure, respectively.

To clarify the underlying mechanisms of the all‐optical control of potentiation and inhibition behaviors, the local phase signals of the CIPS layer were measured by contact resonance piezoresponse force microscopy (PFM). The PFM tip was scanned in situ over the channel region of the MoS_2_/CIPS heterostructure device, while lasers of different wavelengths were simultaneously applied to the top of the device. During the measurement, the voltage was applied between the PFM tip and the gate terminal in order to detect the polarization signal, as illustrated in Figure 2a. The polarization state of the CIPS without light exposure is shown in Figure 2b(i),(iii), which are initialized to the downward polarization (P_down_) and upward polarization (P_up_) states, respectively, by electrical signals. After applying light pulses at different wavelengths (i.e., 660 and 450 nm) for 20 s, most of the polarization in the CIPS region covered by MoS_2_ switched to the upward state under 660 nm illumination (Figure 2b(ii)), whereas it switched to the downward state under 450 nm illumination (Figure 2b(iv)), consistent with the modulation of the channel current in Figure 1f–i. The above results highlight that the polarization direction of CIPS can be selectively switched by tuning the excitation wavelength, indicating a robust, reversible, and bidirectional optical control of ferroelectric states. In addition, as shown in Figure S3, control experiments performed under high‐vacuum conditions exhibit behavior consistent with that observed under ambient conditions. Illumination at 660 and 520 nm induces high‐conductance programming, whereas 450 and 405 nm illumination results in low‐conductance programming. These observations provide evidence that the device operation is not significantly affected by gas adsorption or desorption processes.

To gain deeper insight into this phenomenon, we investigate the underlying physical mechanism responsible for the wavelength‐dependent polarization switching based on the interaction between the photogenerated charges in the MoS_2_/CIPS heterostructure and ferroelectric polarization charges in CIPS. Since the behavior of the devices depends on photon energy relative to the CIPS bandgap, the established carrier dynamic mechanisms are wavelength‐dependent, specifically for excitations below and above the CIPS bandgap. The optical properties of MoS_2_, CIPS, and the MoS_2_/CIPS heterostructure were characterized by absorption and photoluminescence (PL) spectroscopy (Figures S4 and S5). MoS_2_ exhibits characteristic A and B exciton absorption peaks at ∼660 and 610 nm (Figure S4a) and corresponding PL emission at ∼670 and 619 nm (Figure S5), indicating efficient photocarrier generation under the 660 nm excitation used in the optical synaptic experiments. CIPS shows a broad absorption band from ∼250 to 450 nm (Figure S4b) and PL emission centered at ∼450 nm (Figure S5), consistent with its bandgap. Upon formation of the MoS_2_/CIPS heterostructure (Figure S5), the PL of MoS_2_ is significantly quenched, and the PL of CIPS moderately decreases, indicating interfacial charge transfer and carrier redistribution across the vdW interface [37, 38]. These optical characteristics provide a physical basis for the wavelength‐dependent optoelectronic response observed in MoS_2_/CIPS‐based optical synaptic devices. In our metal‐ferroelectric‐semiconductor configuration, the energy band of MoS_2_ is bended upwardly or downwardly at the semiconductor/ferroelectric interface by the down or up polarization of CIPS, respectively. Downward (upward) polarization of CIPS causes accumulation of minority hole (majority electron) carriers at the interface, as illustrated in Figure 2c and Figure S6. Furthermore, to provide direct experimental validation, the surface potential distribution of the MoS_2_/CIPS heterojunction was characterized using Kelvin probe force microscopy (KPFM) under dark conditions as well as under 660 and 450 nm illumination (Figure S7). Based on these measurements, the band alignment and built‐in electric field at the interface, both in the dark and under illumination, were analyzed (Figures S7–S9). As discussed in the Supporting Information (Figures S7 and S8), the work function difference between MoS_2_ and CIPS (ΔW = W_1_–W_2_) is approximately 75 meV. Consequently, upon contact between MoS_2_ and CIPS, a built‐in electric field pointing from CIPS to MoS_2_ is established, accompanied by upward band bending in CIPS and downward band bending in MoS_2_ at the interface (Figure S8b). This behavior is consistent with previously reported studies [39, 40].

In the case of programming with 660 nm light for below‐bandgap excitation (Figure 2d), photocarriers are predominantly generated in MoS_2_. Owing to the built‐in electric field at the heterojunction, the photogenerated carriers are efficiently separated, with photoexcited electrons preferentially transferring from MoS_2_ to CIPS (Figure 2d(i)). The 660 nm illumination corresponds to a photon energy of 1.88 eV, approximately 0.7 eV higher than the bandgap of MoS_2_ (∼1.2 eV), which is sufficient for photoexcited electrons to overcome the conduction band offset between MoS_2_ and CIPS (∼0.3 eV; Figure S8), while remaining insufficient to overcome the much larger valence band offset (∼1.5 eV; Figure S8). This indicates that only hot electron carriers can be transferred to CIPS. As a result, the surface potential of MoS_2_ decreases, whereas that of CIPS increases, leading to an enhanced potential difference of ∼185 mV between CIPS and MoS_2_ (Figure S7b,e). The transferred electrons accumulate at the MoS_2_/CIPS interface and the top surface of CIPS, where they effectively screen the positive ferroelectric polarization charges of CIPS. This enhanced electrostatic screening stabilizes the upward polarization state. When the polarization of CIPS switches from downward to upward (Figure 2d(ii)), electron carriers are more attracted to the interface, increasing the electron concentration in MoS_2_ and therefore the current is increased persistently [41]. The case for exposure to 660 nm light with an initially upward polarization is shown in Figure S10a.

In the case of programming with 450 nm light for above‐bandgap excitations (Figure 2e), a large number of photocarriers are generated in both MoS_2_ and CIPS. In CIPS, photogenerated holes drift toward the MoS_2_/CIPS interface and into the MoS_2_ layer, whereas photogenerated electrons are driven away from the interface due to the upward band bending of CIPS at the heterojunction. Owing to the significantly higher conductivity of MoS_2_ compared to CIPS, the depletion region of the heterojunction is primarily located within the CIPS layer, which strengthens the built‐in electric field that directs hole transport toward the interface. Consequently, the surface potentials of both MoS_2_ (away from the interface) and CIPS (near the interface) decrease, and the potential difference between the CIPS and the MoS_2_ is reduced to ∼15 mV (Figure S7c,f). The resulting accumulation of holes near the interface promotes electrostatic screening of the opposite bound polarization charges, thereby making the downward polarization energetically favorable in this regime. The resulting downward polarization (Figure 2e(ii)) in CIPS will attract hole carriers and therefore the net electron concentration is reduced in MoS_2_, resulting in the lower current. The case for exposure to 450 nm light with an initially downward polarization is shown in Figure S10b. In addition, as shown in Figure S7g, the surface potentials of MoS_2_ stacked on CIPS are extracted from the black solid lines in Figure S7a–c. Compared with the dark condition, the surface potential of MoS_2_ on CIPS increases under 660 nm illumination and decreases under 450 nm illumination. Considering the n‐type nature of MoS_2_, these illumination‐induced potential variations indicate an increased channel conductance under 660 nm illumination and a reduced conductance under 450 nm illumination. This behavior is consistent with the proposed photoresponse mechanism and the electrical measurements of the device, providing direct experimental support for the underlying mechanism. Figure S7h,g further shows that the thicknesses of MoS_2_ and CIPS are approximately 6 and 80 nm, respectively, which are comparable to those used in the device.

In contrast to the other three cases, the IPSC current obtained under 520 nm illumination exhibits increased transient current in the first few seconds, but little difference in the persistent current (as shown in Figure 1f,g), despite the facts that its energy is lower the bandgap of CIPS and more photoexcited carriers can be generated in the MoS_2_ layer at 520 nm. Here, we note that there is a report that illumination at 520 nm can partly generate photoexcited carriers in CIPS [42], indicating that the two mechanisms, which result in the opposite polarization in CIPS, compete with each other in this case. As a result, the below‐bandgap excitation mechanism still governs device behavior in the beginning, but its ability to effectively modulate ferroelectric polarization is comparatively weaker.

### 2.2 All‐Optical Nonvolatile Memory

**FIGURE 3:**
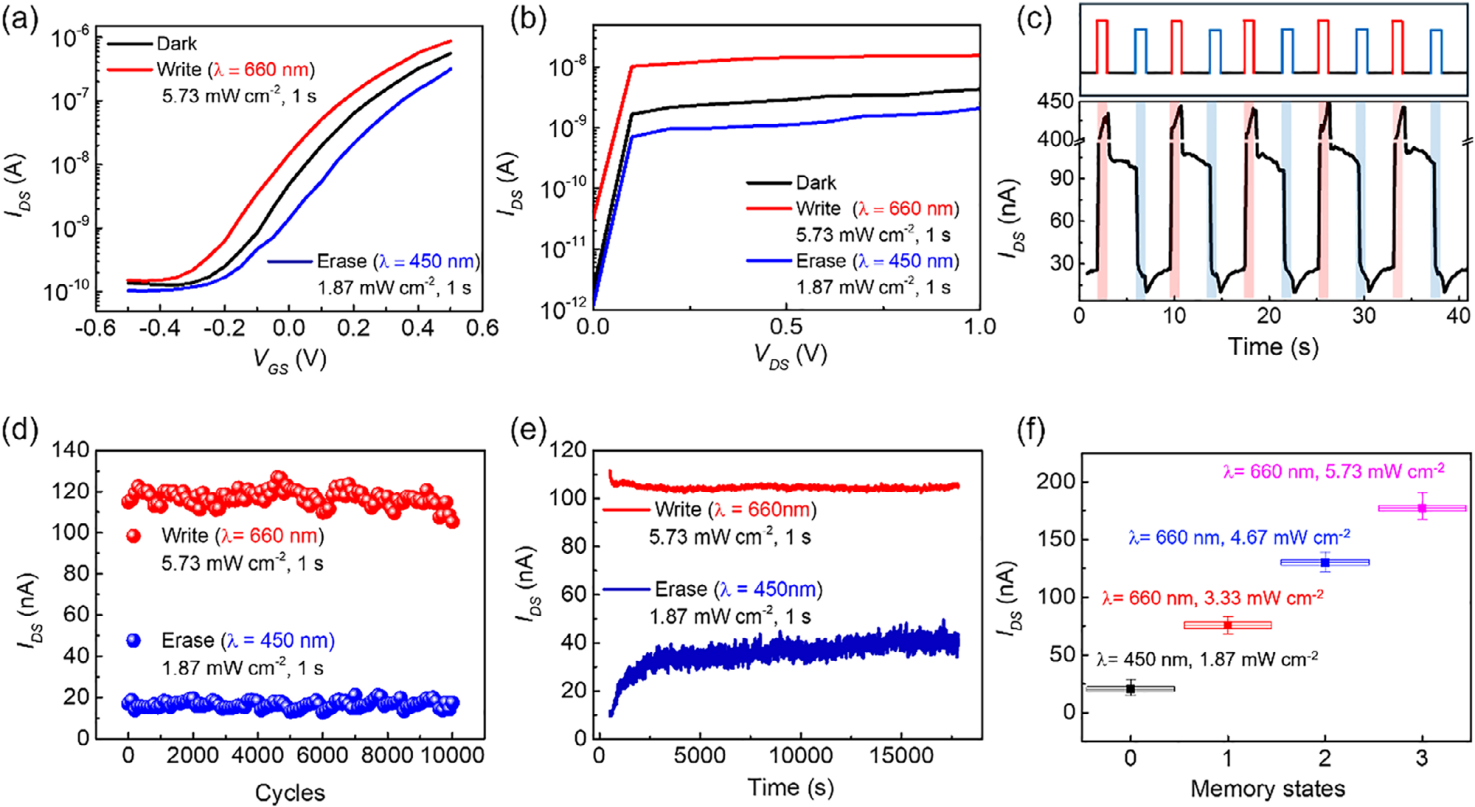
All‐optical nonvolatile memory operation. (a) Transfer curves measured in the dark and after a 1 s exposure to 660 or 450 nm light under a constant *V_DS_* of 0.5 V. (b) Output characteristics measured in the dark and after a 1 s illumination with 660 or 450 nm light without the gate bias. (c) Dynamic memory operation of the optical‐FeFET in all‐optical mode. (d) Endurance and (e) retention characteristics of the optical‐FeFET. (f) Optical multilevel memory achieved by tuning the power density. Error bars represent statistical variation obtained from multi‐cycle and multi‐device measurements.

By exploiting the above mechanism, the MoS_2_/CIPS heterostructure can be extended beyond all‐optical nonvolatile memory to function as optoelectronic synapses and logic‐in‐memory devices, providing a versatile platform for neuromorphic and in‐memory computing applications. The transfer curve of the optical‐FeFET exhibits a typical counterclockwise hysteresis induced by ferroelectricity, as shown in Figure S11. The conductance states of the MoS_2_/CIPS‐based optical‐FeFET, regulated by optical signals as presented in the *I_DS_*–*V_GS_* (Figure 3a) and *I_DS_*–*V_DS_* (Figure 3b) characteristics, enable programming (optical‐write) and erasing (optical‐erase) operations by simple illumination with 660 and 450 nm wavelengths, respectively. In Figure 3a, the transfer curves were measured in the dark and after a 1 s exposure to either 660 nm (5.73 mW cm^−2^) or 450 nm (1.87 mW cm^−2^) light under a constant *V_DS_* of 0.5 V. In Figure 3b, output characteristics were measured in the dark and after a 1 s illumination with 660 or 450 nm light, revealing two distinct resistance states with an on/off ratio exceeding 10 when *V_DS_* was 0.5 V. The conductance modulation under four wavelengths (660, 520, 450, and 405 nm) further confirmed optical‐write under longer wavelengths (660 and 520 nm) and optical‐erase under shorter wavelengths (450 and 405 nm) (Figure S12), consistent with the PSC responses in Figure 1f–i. Figure 3c demonstrates the dynamic memory operation of the optical‐FeFET in all‐optical mode. The top panel illustrates the optical pulse schemes for the write and erase functions, where 1 s pulses of 660 and 450 nm light were applied, respectively. Optical‐write induced a positive photocurrent, driving the device into a low‐resistance state (LRS), whereas optical‐erase produced a negative photocurrent, restoring the device to its initial high‐resistance state (HRS). A constant *V_DS_* of 0.5 V was used for the read operation without the gate bias. Threshold voltage shifts induced by the four laser wavelengths are shown in the transfer characteristics in Figure S13, further confirming the positive photocurrent response under 660 and 520 nm illumination and the negative photocurrent response under 450 and 405 nm illumination.

The reliability of the optical‐FeFET was further verified through cyclic endurance testing, where alternating optical‐program and optical‐erase cycles were applied (Figure 3d), confirming the stability of both LRS and HRS over 10^4^ cycles. Additionally, a retention test was performed after applying a 1 s pulse of 660 or 450 nm light (Figure 3e), showing that the two resistance states remained clearly distinguishable for over 10^4^ s. Furthermore, multilevel optical memory corresponding to 2‐bit storage (four distinct conductance states) was realized by modulating the power density of the optical write operation. Optical erasing under 450 nm illumination defined state “0”, while optical writing at 660 nm with power densities of 3.33, 4.67, and 5.73 mW cm^−2^ generated states “1”, “2”, and “3”, respectively (Figure 3f). The reliability and uniformity of the multilevel operation were evaluated through repeated optical programming over 500 cycles on the same device, together with single‐cycle measurements from five devices under identical conditions (Figure S14). In Figure 3f, each data point represents the averaged conductance value, with error bars indicating the maximum and minimum values and boxes denoting the standard deviation. The conductance separation between adjacent levels is larger than the statistical variation, resulting in non‐overlapping distributions and confirming the robustness and distinguishability of all intermediate memory states. Compared with previous optical nonvolatile memory work based on ferroelectric devices (Table S3) [9], our device achieves fully all‐optical nonvolatile memory with superior retention and endurance, while prior devices required optical writing combined with electrical erasing. Although a larger on/off ratio was achieved in that study, it required higher optical power densities and longer pulse widths.

### 2.3 All‐Optical Artificial Synaptic Properties and Bio‐Inspired Behavior Simulation

**FIGURE 4:**
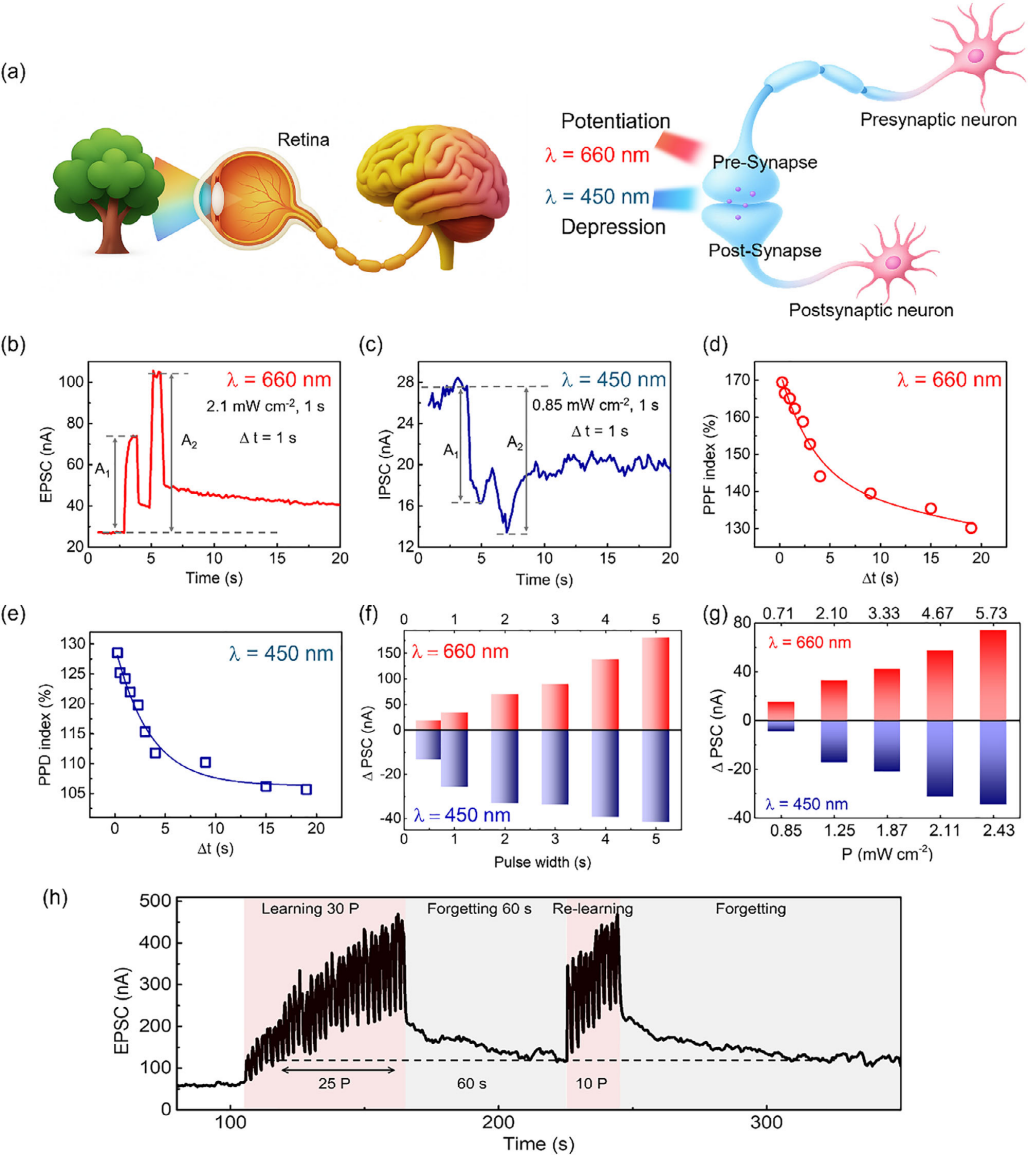
All‐optical artificial synaptic properties. (a) Illustration of retina‐inspired optoelectronic synaptic functions for artificial visual systems. (b), (c) Paired‐pulse facilitation (PPF) and paired‐pulse depression (PPD) responses induced by two consecutive light pulses. (d), (e) Inter‐spike interval (∆t) dependence of PPF and PPD indices fitted with a double exponential decay function. (f) Light duration‐dependent (0.5, 1, 2, 3, 4, and 5 s) variation of postsynaptic current changes (∆PSC) under 660 and 450 nm light. (g) Light intensity‐dependent ∆PSC (660 nm: 0.71, 2.1, 3.33, and 5.73 mW cm^−2^; 450 nm: 0.85, 1.25, 1.87, 2.11, and 2.43 mW cm^−2^). Pulse width = 1 s. (h) Human learning‐experience behavior.

In addition to demonstrating stable nonvolatile memory performance, we investigate all‐optical synaptic plasticity in the optical‐FeFET. Owing to the wavelength‐dependent photocurrent response, red light (660 nm) induces potentiation, whereas blue light (450 nm) triggers depression, enabling retina‐inspired optoelectronic neuromorphic computing suitable for artificial visual systems, as illustrated in Figure 4a. Paired‐pulse measurement is implemented in the optical‐FeFET, including paired‐pulse facilitation (PPF) and paired‐pulse depression (PPD). When two consecutive presynaptic spikes are applied, the second light pulse induces a larger conductance change due to incomplete carrier relaxation during the inter‐spike interval (∆t). This paired‐pulse facilitation/inhibition (PPF/PPD) exemplifies short‐term plasticity (STP), emulating transient synaptic modifications essential for short‐term memory (STM) [43]. Figure 4b,c presents the PPF and PPD responses induced by two consecutive light pulses (pulse width = 1 s, ∆t = 1 s) at 660 (2.1 mW cm^−2^) and 450 nm (0.85 mW cm^−2^), respectively. The PPF/PPD index is defined as

$$\mathrm{PPF}\;\mathrm{or}\;\mathrm{PPD}\;\mathrm{Index}=\frac{A_{2}}{A_{1}}\;\times \;100\%$$

where A_2_ and A_1_ are the postsynaptic currents induced by the first and second pulses. The maximum PPF and PPD values at a 250 ms interval reach approximately 170% and 128%, respectively. The inter‐spike interval (∆t) dependence of PPF and PPD index, plotted in Figure 4d,e, can be well fitted with a double exponential decay function:

$$\mathrm{PPF}\;\mathrm{or}\;\mathrm{PPD}\;\mathrm{Index}=C_{1}\;\exp\left(-\frac{x}{t_{1}}\right)+C_{2}\exp\left(-\frac{x}{t_{2}}\right)+y_{0}$$

where t_1_ and t_2_ are characteristic relaxation times and C_1_ and C_2_ are the initial facilitation magnitudes. The values of t_1_ and t_2_ are 1.04 and 3.39 s, respectively, in the case of EPSC, whereas for IPSC, they are 3.5 and 2.07 s, respectively.

The frequency, intensity, number, and duration of external stimuli critically determine synaptic modulation, producing either transient or persistent changes in conductance. These dynamics manifest as STP and long‐term plasticity (LTP), both essential for biological learning. The STP reflects temporary modifications associated with short‐term memory (STM), whereas LTP represents enduring changes that consolidate STM into long‐term memory (LTM) by reinforcing synaptic connections [44]. In artificial synaptic devices, achieving this transition by adjusting light‐pulse parameters is essential for mimicking the hierarchical learning processes of the brain. The light duration‐dependent and intensity‐dependent responses of the device are summarized in Figures S17 and S18, with corresponding changes in postsynaptic current (∆PSC, recorded 10 s after light irradiation) shown in Figure 4f,g. Both EPSC and IPSC amplitudes change increase monotonically with longer pulse durations (0.5–5 s) or higher power intensities at 660 and 450 nm, respectively. Specifically, ∆PSC increases from 17.81 to 180.6 nA under 660 nm and from −13.2 to −41.71 nA under 450 nm with increasing pulse duration (Figure 4f), exhibiting a similar trend with increasing power intensity (Figure 4g). In addition, to further elucidate the transition from short‐term memory (STM) to long‐term memory (LTM), time‐resolved photocurrent measurements were performed using short optical pulses, followed by an analysis of the post‐illumination relaxation dynamics (as shown in Figures S15 and S16). The temporal evolution of the photocurrent after optical excitation was fitted with a bi‐exponential model, enabling the extraction of characteristic relaxation time constants associated with distinct carrier relaxation processes. Under 660 nm illumination (Figure S15), increasing the pulse duration from 100 to 500 ms leads to a pronounced increase in the slow relaxation time constant (τ_2_) from 12.87 to 171.72 s (Figure S15e), indicating a transition from a short‐lived relaxation regime to a long‐lived, stabilized conductance state. This abrupt extension of τ_2_ provides kinetic evidence for the transition from STM‐like to LTM‐like behavior. Under 450 nm illumination (Figure S16), when the pulse duration is increased from 500 ms to 1 s, the extracted τ_2_ increases dramatically to a value far exceeding the experimental observation window (τ_2_ ≫ measurement timescale) (Figure S16b,c). This apparent divergence of τ_2_ indicates the formation of a stationary interfacial charge configuration, stabilized by cumulative ferroelectric polarization. Furthermore, this time‐resolved photocurrent transients at both wavelengths reveal two relaxation time scales, corresponding to fast interfacial carrier redistribution (τ_1_) and slower polarization‐coupled relaxation (τ_2_). While τ_1_ is weakly dependent on pulse duration and governed by intrinsic interfacial fields, τ_2_ strongly increases with excitation duration and wavelength, indicating progressive carrier stabilization mediated by ferroelectric polarization at the MoS_2_/CIPS interface. Beyond duration‐ and intensity‐dependent plasticity, higher‐order learning rules, including spike‐number‐dependent plasticity (SNDP) and spike‐frequency‐dependent plasticity (SFDP), were also demonstrated, as shown in Figures S19 and S20, further confirming the device's ability to emulate complex synaptic learning behaviors.

Furthermore, the device replicates human learning‐experience behavior under 660 nm light stimulation, characterized by four sequential processes: learning, forgetting, relearning, and secondary forgetting (Figure 4h). During the first learning cycle, 30 light pulses (1 s duration, 0.5 Hz frequency) induce a gradual EPSC increase, followed by decay upon light removal, representing forgetting. After a 60 s forgetting interval, the learning level corresponding to 25 pulses in the initial cycle is recovered with only 10 pulses during relearning, demonstrating memory retention. Notably, the subsequent forgetting process occurs at a significantly slower rate, reflecting the facilitation effect of prior learning experience.

**FIGURE 5:**
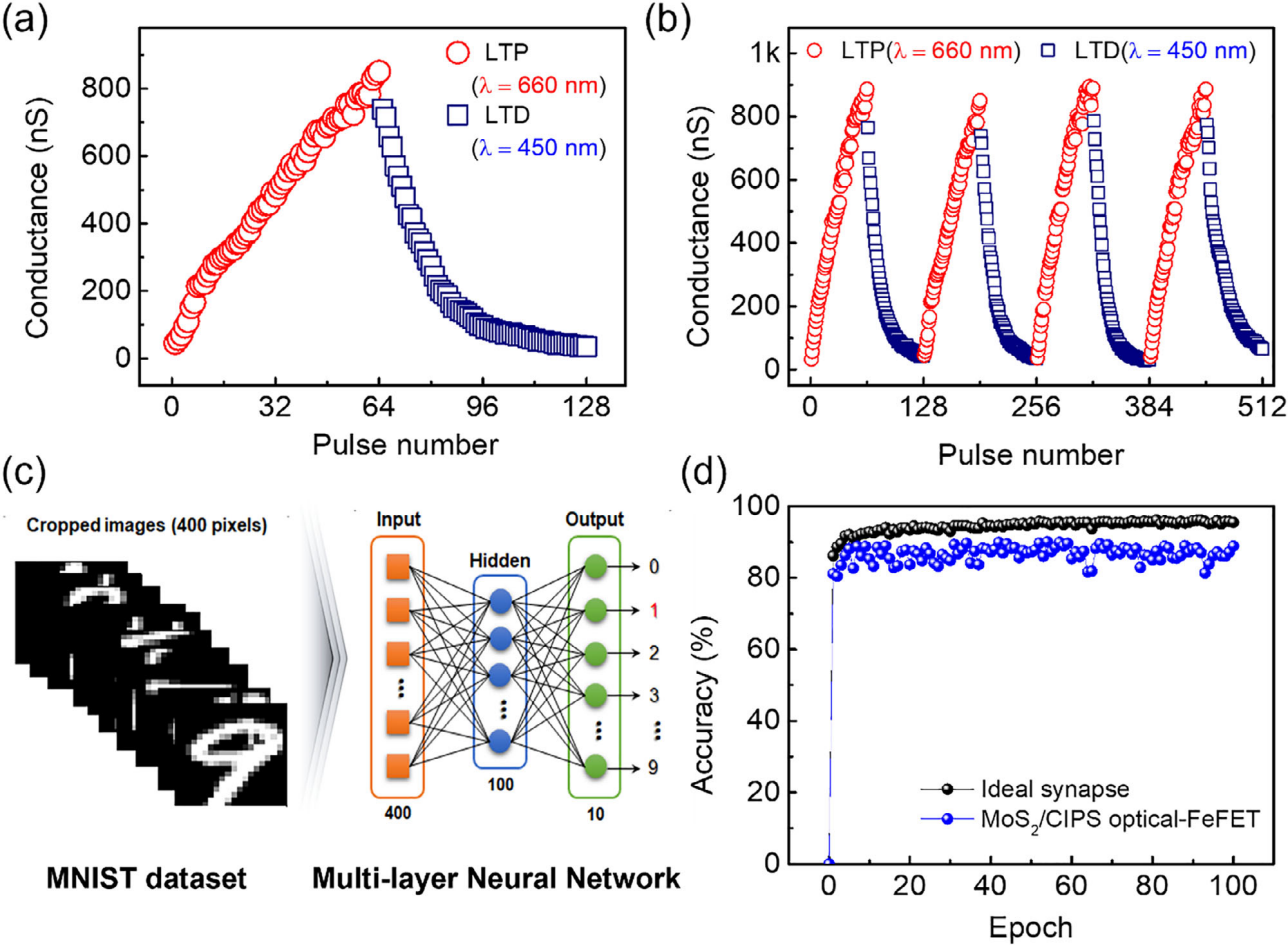
Artificial neural network (ANNs) simulation. (a) Long‐term potentiation (LTP: 660 nm) and long‐term depression (LTD: 450 nm) realized in an all‐optical mode. (b) Four consecutive LTP/LTD optical cycling tests. (c) Schematic of a three‐layered ANN model for recognizing handwritten digit images based on the optical‐FeFET. (d) Image recognition accuracy over 100 training epochs using the MNIST database.

Long‐term synaptic plasticity, comprising LTP and LTD of synaptic weights, plays a significant role in regulating memory formation in hippocampal neurons [44]. Unlike previously reported artificial synaptic devices that require combined optical and electrical pulses [7, 9, 31, 33, 34, 35, 43, 44, 45], our optical‐FeFET achieves LTP and LTD exclusively through optical stimulation. As shown in Figure 5a, long‐term potentiation (660 nm, 2.1 mW cm^−2^) and depression (450 nm, 0.85 mW cm^−2^) are realized in all‐optical mode using 64 pulses (1 s duration, 0.5 Hz frequency). The dynamic range, defined by the conductance ratio G_max_/G_min_, reaches approximately 24.2, confirming robust modulation of synaptic weights. Linearity fitting of the weight updates is presented in Figure S21, with results from other wavelength combinations provided in Figure S22. Furthermore, Figure 5b demonstrates reproducible and reversible LTP/LTD behavior across four consecutive optical cycling tests, highlighting the stability of the all‐optical modulation. After approximately six months of storage under ambient conditions without encapsulation, the MoS_2_/CIPS device retains its electrical characteristics and light‐induced synaptic plasticity, with only a slight reduction in nonlinearity, demonstrating its environmental robustness (Figure S23, Table S4).

To further evaluate the practical applicability of the demonstrated all‐optical synaptic plasticity, we performed artificial neural network (ANN) simulations using the NeuroSim+ framework. The experimentally obtained device characteristics, including linearity and dynamic range, were incorporated into the model, with the conductance state of the device mapped as the synaptic weight, analogous to biological synapses. The continuous and nearly linear modulation of current enabled realistic training. Nonlinearity factors extracted from potentiation, depression, and dynamic range were used as input to the simulator to evaluate the pattern recognition performance on the MNIST handwritten digit dataset. Detailed device parameter extraction, including nonlinearity factors and dynamic range, is presented in Figure S21.

A multilayer perceptron (MLP) comprising 400 input neurons, 100 hidden neurons, and 10 output neurons was implemented, where the 400 inputs corresponded to pixel intensities of 20 × 20 grayscale images and the 10 outputs represented digits 0–9 (Figure 5c). The network was trained on a randomly selected subset of 8000 images from the 60 000‐image training dataset and tested on an independent set of 10 000 images. As shown in Figure 5d, an ideal synapse achieved a recognition accuracy of approximately 96% after 100 training epochs, while the device‐based synapse maintained an accuracy exceeding 90%, demonstrating its potential for practical neuromorphic computing applications. Additional simulations with a larger network (400 input neurons, 300 hidden neurons, 12 000 training samples per epoch) show an ∼1% improvement in recognition accuracy (Figure S24). Importantly, all recent reports of all‐optical synapses use similar ANN configurations (Table S5), yet they achieve lower or comparable accuracy, whereas our device reaches 91.89%, demonstrating superior or comparable performance. Notably, the second‐scale optical programming timescale does not impose a limitation on the ANN implementation in this work. Synaptic weights are programmed only during the training (or calibration) stage, after which ANN inference is performed electronically using fixed, nonvolatile weight states. Consequently, the inference speed is governed by the electronic readout circuitry rather than by the optical switching time of the device. Therefore, the optical programming timescale does not affect the inference throughput or operational performance of the ANN. Furthermore, the second scale switching time originates from low‐power optical operation rather than a fundamental device limitation, reflecting a trade‐off between switching speed and energy consumption. Increasing the optical power density enables switching with pulse widths down to 1 ms (Figure S25), extending device operation to the millisecond timescale. A comparison of reported all‐optical artificial synapses is provided in Table S1. In addition, LTP/LTD synaptic plasticity was examined in multiple devices with different MoS_2_ and CIPS thicknesses (Figures S26 and S27). As summarized in Table S6, all devices exhibit consistent synaptic behavior, with only modest variations in linearity.

### 2.4 All‐Optical Reconfigurable Logic‐in‐Memory

To establish a versatile platform for neuromorphic and in‐memory computing, we further investigated reconfigurable logic‐in‐memory (LiM) operations in an entirely optical mode. As described above, the device polarization can be continuously and reversibly modulated by the combined influence of 660 and 450 nm light, enabling dynamic control of the memory state. Consequently, all‐optical reconfigurable logic gates were demonstrated within a single device.

**FIGURE 6:**
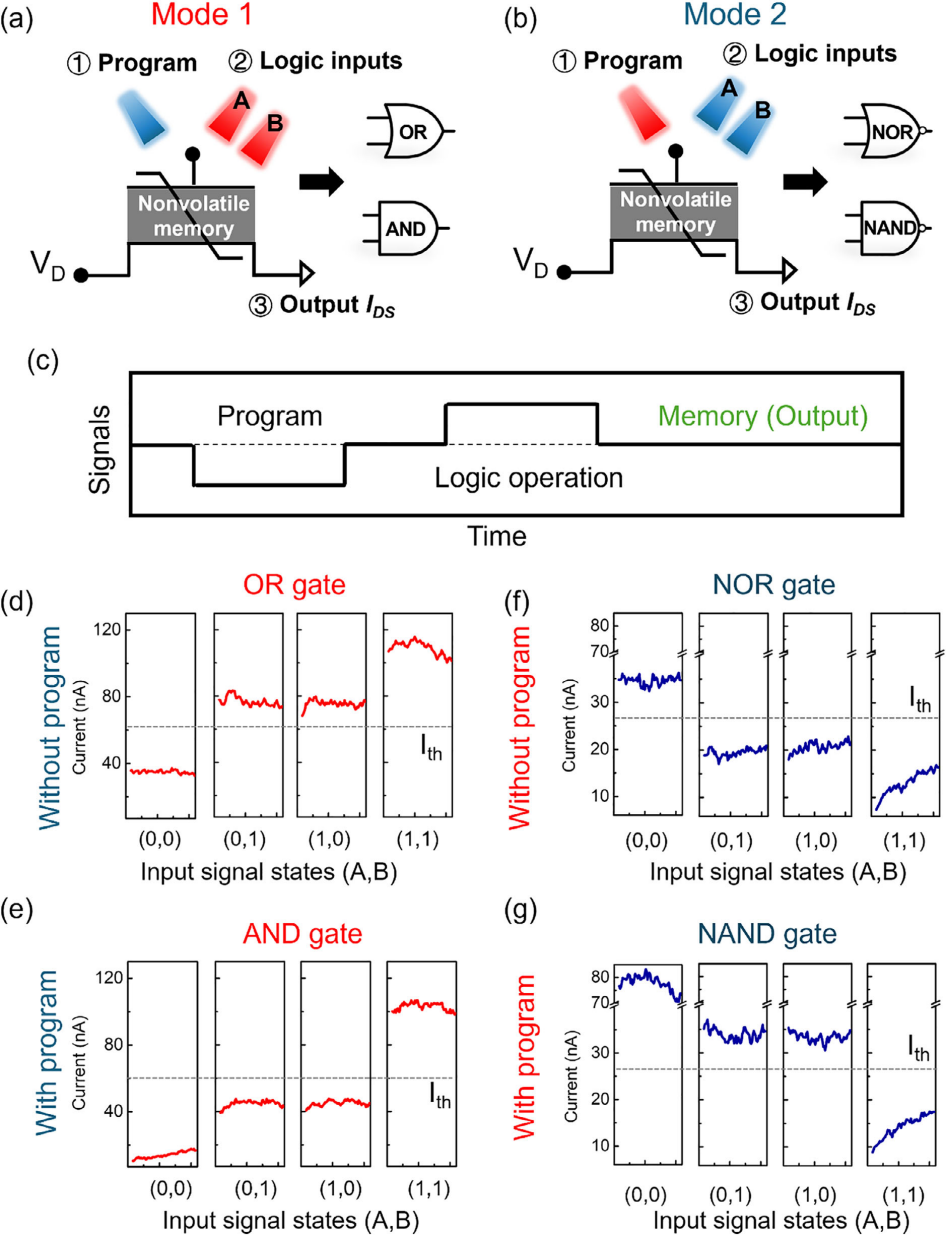
All‐optical logic‐in‐memory application and its reconfigurability. (a), (b) Schematic of reconfigurable all‐optical logic‐in‐memory operation when 660 nm (a) or 450 nm (b) light is used as the logical input signal. (c) Illustration of the operation process adopted to achieve reconfigurable logic operations. (d–g) *I_DS_* vs. time for four all‐optical Boolean logic functions (OR, AND, NOR, and NAND) performed using a single optical‐FeFET with or without Pre‐programming signals.

As shown in Figure 6a,b, one wavelength serves as the logic input while the other acts as a pre‐programming signal; by alternating the pre‐program signal, the device can implement distinct Boolean logic functions. In mode 1, two 660 nm lights serve as logic inputs, while a 450 nm light functions as the pre‐programming signal. In mode 2, two 450 nm lights serve as logic inputs, with a 660 nm light acting as the pre‐programming signal. Figure 6c depicts the operational procedure for mode 1 and mode 2, respectively. Pre‐program is first performed using a 1 s laser pulse at either 450 or 660 nm (light on = with pre‐program, light off = without pre‐program) to set the initial polarization and conductance of the device. Logical inputs A and B are then applied using two separate light sources of either 660 or 450 nm simultaneously (light on = input “1”, light off = input “0”), and the output current (*I_DS_*) is measured to determine the logic result, with representative data presented in Figure 6d–g. When 660 nm light is used as the two logic inputs (Figure 6d,e), without a 450 nm pre‐program (Figure 6d), the initial conductance is higher than with 450 nm pre‐program case. The output current exceeds the threshold current (I_th_) (“1”) when any 660 nm input is “1” and falls below the I_th_ (“0”) only when both 660 nm inputs are “0”, thereby implementing an OR gate. With a 450 nm pre‐program (Figure 6e), the initial conductance is lower than without 450 nm pre‐program case, and the output reaches the high conductance state (above I_th_) only when both 660 nm inputs are “1”, thereby realizing an AND logic gate. Furthermore, when 450 nm light is used as the two logic inputs (Figure 6f,g), without a 660 nm pre‐program (Figure 6f), the initial conductance is lower than with 660 nm pre‐program case. The output current exceeds the I_th_ (“1”) only when both 450 nm inputs are “0” and drops below I_th_ (“0”) if any 450 nm input is “1”, thereby performing a NOR logic gate. With a 660 nm pre‐program (Figure 6g), the initial conductance is higher than without the 660 nm pre‐program case, and the output current falls below the I_th_ (“0”) only when both inputs are “1”, executing a NAND logic gate. To reliably distinguish output states, the I_th_ is set at 25 nA for NOR and NAND operations when both inputs are 450 nm, and at 50 nA for OR and AND gates when both inputs are 660 nm. This consistent thresholding enables dependable discrimination of logic functions solely based on optical input conditions. Different logic functions are realized using two predefined reference current thresholds associated with distinct optical programming conditions. In cascaded operation, the output current is converted back into an optical signal, during which the optical intensity can be fully regenerated once the reference threshold is exceeded, preventing the accumulation of base‐level current variations across successive logic stages. The corresponding truth tables, including one pre‐program signal, two input logic signals (A and B), and the output, are reported in Figure S28. Statistical analysis over 50 operation cycles shows that the output current distributions of the four Boolean logic gates are separated for logic “0” and logic “1” states (Figures S29 and S30). Fixed current thresholds of 60 nA (Mode 1: OR/AND) and 27 nA (Mode 2: NOR/NAND) ensure that the maximum logic “0” current remains below, while the minimum logic “1” current stays above the threshold across all cycles, providing an error margin for reliable logic.

## 3 Conclusion

This study demonstrates, for the first time, bidirectional polarization switching in a vdW heterostructure composed of ferroelectric CIPS and semiconducting MoS_2_. The switching mechanism is attributed to the redistribution of photo‐induced carriers and screening charges at the interface, supported by two wavelength‐dependent carrier dynamic mechanisms. High‐performance all‐optical nonvolatile memory was achieved, exhibiting an on/off ratio of the memory state exceeding 10, retention over 10^4^ s, endurance beyond 10^4^ cycles, and multilevel cell storage enabled via light intensity modulation. Moreover, the device emulates retina‐inspired synaptic plasticity and integrates sensing, processing, and memory within a single architecture. Furthermore, it demonstrates purely optical reconfigurable Boolean logic gates (AND, OR, NAND, NOR) within a single device. Collectively, these results not only advance versatile platforms for fully optical in‐memory computing devices but also open new opportunities for scalable, energy‐efficient optoelectronic computing and integrated intelligent systems.

## 4 Methods

### 4.1 Device Fabrication

The bottom‐gate terminal was fabricated via electron‐beam evaporation following electron‐beam lithography. Bulk MoS_2_ (2D Semiconductors) and CIPS (HQ Graphene) crystals were mechanically exfoliated, and thin flakes were transferred onto the bottom gate using a dry‐transfer method to form a heterostructure. Source and drain electrodes were defined using electron‐beam lithography and subsequently deposited with Ti/Au (10/80 nm) via electron‐beam evaporation.

### 4.2 Device and Material Characterization

An optical microscope (Olympus, BX51M) was employed to confirm the size and morphology of the flakes and devices. The thickness of the thin flakes was measured using atomic force microscopy (AFM; Park‐NX10, M/s Park Systems Corp.) in noncontact mode. Contact resonance PFM measurements were performed using the same AFM system in contact mode. The frequency and amplitude of the alternating voltage (Vac) applied for PFM imaging were 287 kHz (contact resonance frequency) and 0.5 V, respectively. Raman spectroscopy was performed using a Raman spectrometer (WITEC Alpha 300 m) with a 532 nm excitation laser. All electrical characterizations were conducted using a Keithley 4200 semiconductor characterization system (M/s Tektronix Inc.) at room temperature. During electrical characterization, the device was maintained under ambient atmospheric conditions. Light pulses were generated using a true waveform generator (Keysight 33600A). A power meter (Ophir Photonics) was used to measure the light intensity.

## Conflicts of Interest

The authors declare no conflicts of interest.

## Supporting information




**Supporting file**: advs74170‐sup‐0001‐SuppMat.docx

## Data Availability

The data that support the findings of this study are available from the corresponding author upon reasonable request.
