# Supplementary material for: All‐Optical Control of Bidirectional Polarization Switching in Ferroelectric Heterostructures for Neuromorphic and In‐Memory Computing
Source: Adv Sci (Weinh). 2026 Jan 29;13(20):e22092. doi: 10.1002/advs.202522092 (PMC13067862; doi:10.1002/advs.202522092)
Supplement: Supplementary file 1 — Supporting file: advs74170‐sup‐0001‐SuppMat.docx [file ADVS-13-e22092-s001.docx]

Supporting Information

**All-Optical Control of Bidirectional Polarization Switching in Ferroelectric Heterostructures for Neuromorphic and In-Memory Computing**

*Jingjie Niu^1,2^, Jiahui Lyu^1,2^, Jie Li^3^, Koyal Suman Samantaray^1,2^, Cheolhwa Jang^1,2^, Yoonmyung Lee^4^, Ji-Sang Park^1,2,5 *^, and Sungjoo Lee^1,2,5 *^*

^1^SKKU Advanced Institute of Nanotechnology (SAINT), Sungkyunkwan University, Suwon 16419, Korea

^2^Department of Nano Science and Technology, Sungkyunkwan University, Suwon 16419, Korea

^3^IMEC, Kapeldeef 75, 3001 Heverlee, Belgium

^4^Department of Electrical and Computer Engineering, Sungkyunkwan University, Suwon 16419, Korea

^5^Department of Nano Engineering, Sungkyunkwan University, Suwon 16419, Korea

**Table S1.** Comparison of all-optical artificial synapse and logic-in-memory.


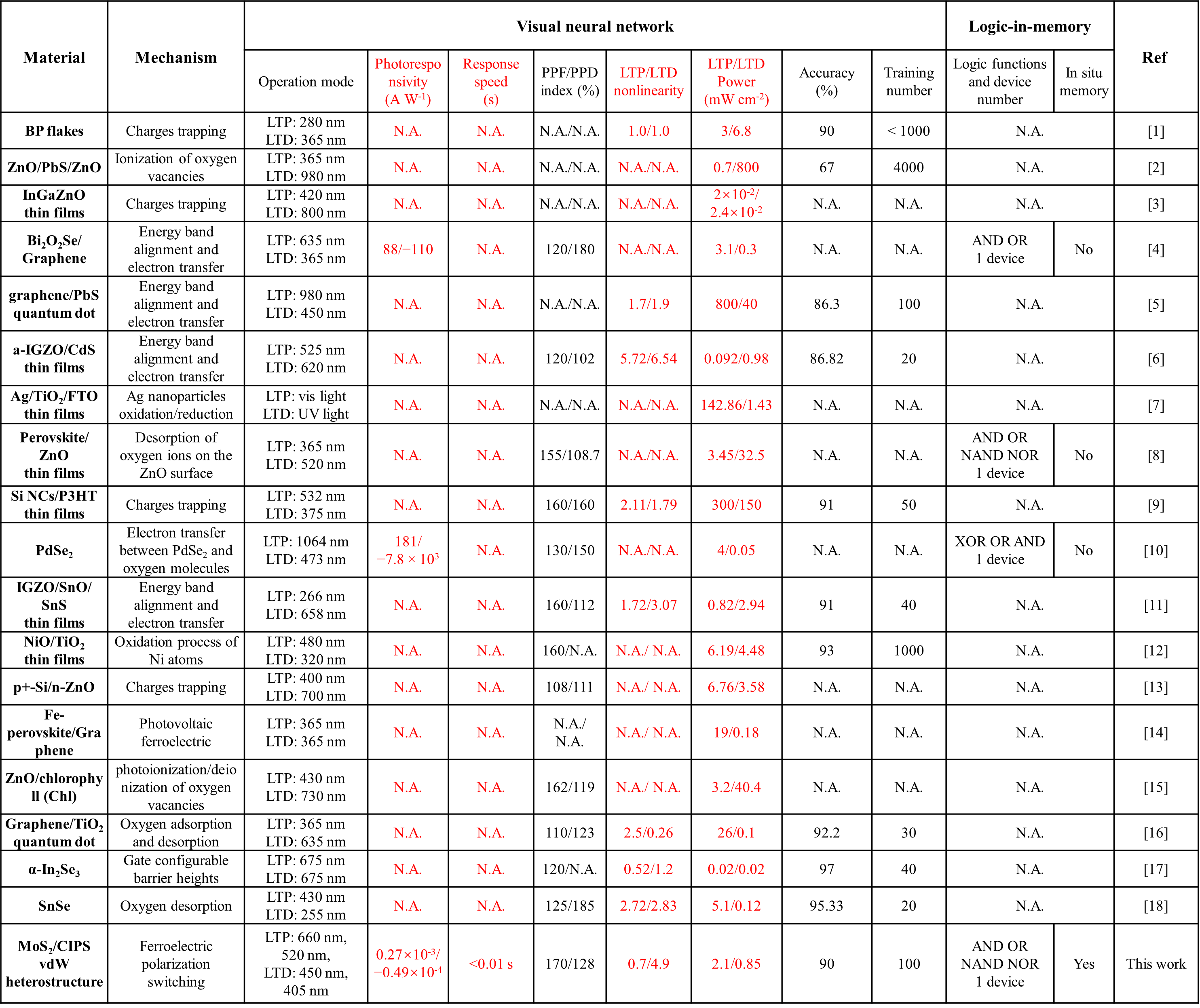


Recent efforts toward all-optical neuromorphic devices have explored diverse material platforms and mechanisms. For instance, black phosphorus (BP) with surface oxidation layers,^[1]^ 2D ferroelectric semiconductors such as α-In_2_Se_3_ with gate-tunable barrier heights,^[17]^ and heterostructures composed of organic polymers or inorganic oxide semiconductors ^[2–13,15,16,18]^ have been employed to achieve all-optical plasticity via energy band alignment and carrier transfer, light-induced interfacial carrier trapping, or defect-state modulation. Nevertheless, several challenges persist: BP suffers from environmental instability and limited device performance; α-In_2_Se_3_ phototransistors still require electrical assistance; and semiconductor heterostructure-based optical synapses often exhibit volatile responses and poor nonlinearity control due to metastable trap states. Furthermore, only a few studies have investigated the integration of logic operations, which is essential for constructing functional computing systems. Collectively, these limitations significantly hinder scalability and practical deployment in large-scale neuromorphic hardware.

In this study, we propose an alternative strategy based on all-optical control of bidirectional ferroelectric switching with intrinsic nonvolatility. Our optical-FeFET demonstrates a broad operational wavelength range from the visible to near-UV spectrum, robust synaptic plasticity with large paired-pulse facilitation (PPF) and paired-pulse depression (PPD) indices of 170% and 128%, respectively, high image recognition accuracy obtained by Artificial neural network (ANNs) simulation, and reconfigurable all-optical logic-in-memory capability, enabling multiple Boolean logic gates within a single device. Collectively, these results establish a universal light-controlled in-memory computing platform that seamlessly integrates artificial synaptic functionalities with reconfigurable logic operations.

Table S2. Qualitative comparison of key features with representative all-optical and hybrid ferroelectric devices.

| Materials | Mechanism | Operation mode | Optical bidirectionality (reversibility) | Optical nonvolatile memory | Wavelength-selective control | Ref |
| --- | --- | --- | --- | --- | --- | --- |
| α-In_2_Se_3_ | Ferroelectric polarization switching | Hybrid | No | Yes | No | ^[25]^ |
| ReS_2_/hBN/CuInP_2_S_6_ | Ferroelectric polarization switching | Hybrid | No | No | No | ^[26]^ |
| SnS_2_/h-BN/CuInP_2_S_6_ | Ferroelectric polarization switching | Hybrid | No | No | No | ^[27]^ |
| WS_2_/PZT | Ferroelectric polarization switching | Hybrid | No | No | No | ^[28]^ |
| CIPS/Gr/h-BN/Te | Ferroelectric polarization switching | Hybrid | No | No | No | ^[29]^ |
| α-In_2_Se_3_ | Gate configurable barrier heights | All-optical | No | No | No | ^[17]^ |
| Fe-perovskite/Graphene | Photovoltaic ferroelectric | All-optical | No | No | No | ^[14]^ |
| MoS_2_/CIPS  vdW heterostructure | Ferroelectric polarization switching | All-optical | Yes | Yes | Yes | This work |

Table S2 indicates that previously reported hybrid photo-electrical ferroelectric devices generally exhibit only one-directional optical programming or require an external electrical bias to enable reverse switching. In contrast, our device realizes fully optical, reversible, wavelength-selective polarization switching with nonvolatile retention, a combination of functionalities that has not been demonstrated in prior studies.

Table S3. Benchmarking key metrics of the present device versus representative all-optical and hybrid ferroelectric devices.

| Materials | Operation  mode | On/off  ratio | Retention | Endurance | Writing/erasing energy consumption | Switching speed  (pulse width) | Ref |
| --- | --- | --- | --- | --- | --- | --- | --- |
| α-In_2_Se_3_ | Hybrid | 10^4^ | 5000 s | 800 | Writing (optical): 1.22 J cm^-2^  Erasing (electrical): 200 mJ | Optical: 11 s  Electrical: 5 s | ^[25]^ |
| MoS_2_/CIPS  vdW heterostructure | All-optical | 10 | 10^4^ s | 10^4^ | Writing (optical): 5.73 mJ cm^-2^  Erasing (optical): 1.87 mJ cm^-2^ | Optical: 1 s | This work |

As summarized in Table S3, compared with the reported device,^[25]^ our device shows all-optical nonvolatile memory with improved retention (>10^4^) and endurance (10^4^) whereas the reported work relies on optical writing combined with electrical erasing. Although a larger on/off ratio was achieved in that study, it required higher optical power densities and longer pulse widths, resulting in a slower switching speed.


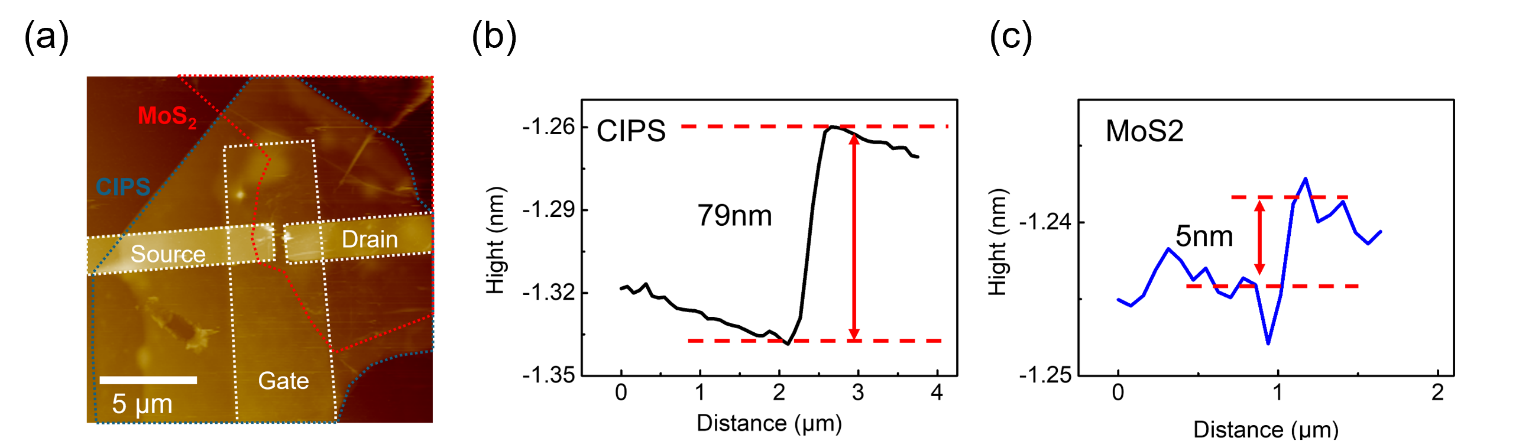
Figure S1. AFM image and height profiles of the optical-FeFET. (a) AFM image of the optical-FeFET device. Scale bar = 5 μm. (b) Thickness of the CIPS flake (measured along the dotted line in (a)) is approximately 79 nm. (c) Thickness of the MoS_2_ flake (red line) is approximately 5 nm.

Figure S1a presents the AFM images and height profiles of the optical-FeFET. The thicknesses of CIPS and MoS_2_ were determined to be 79 nm (Figure S1b) and 5 nm (Figure S1c), respectively.


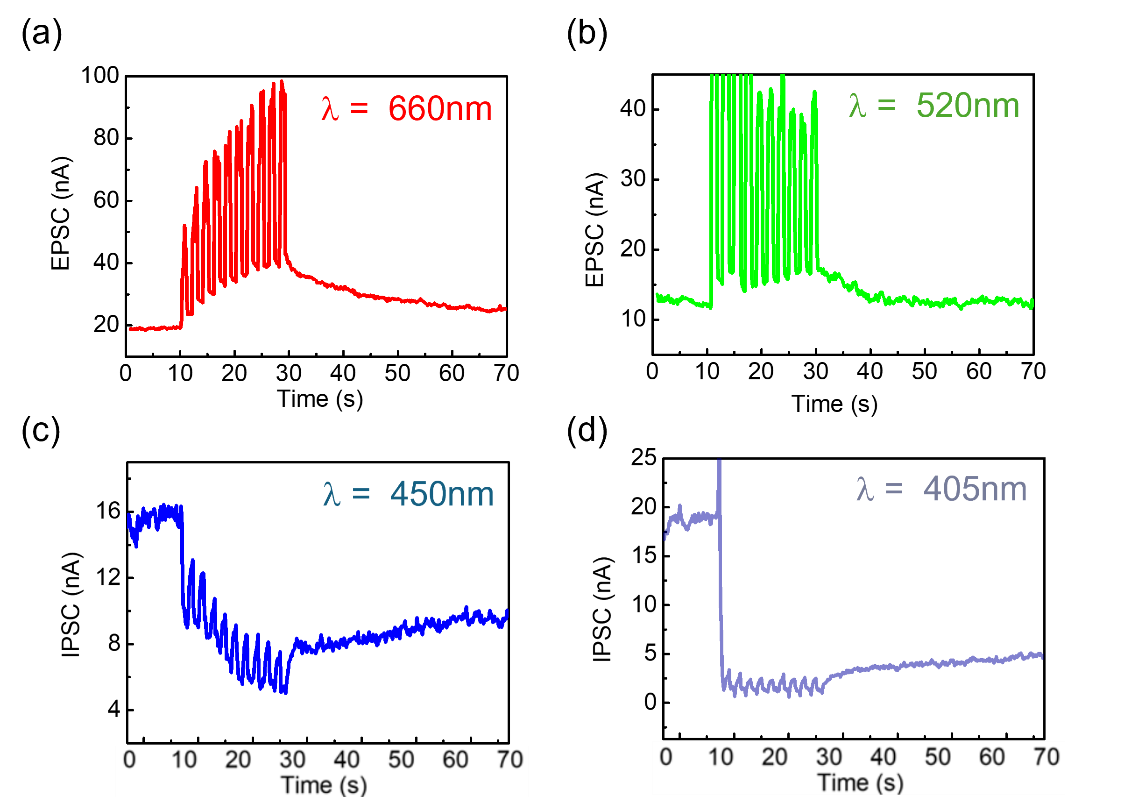


Figure S2. PSC responses of the device at four different wavelengths. (a) 660 nm, (b) 450 nm, (c) 450 nm, and (d) 450 nm.

The PSC responses under ten consecutive optical pulse stimulations at four wavelengths are presented in Figures S4a–S4d, confirming the reproducibility and stability of the wavelength-dependent synaptic plasticity. Specifically, 660- and 520-nm light induce potentiation, while 450 and 405 nm light induce depression in a single optical-FeFET.


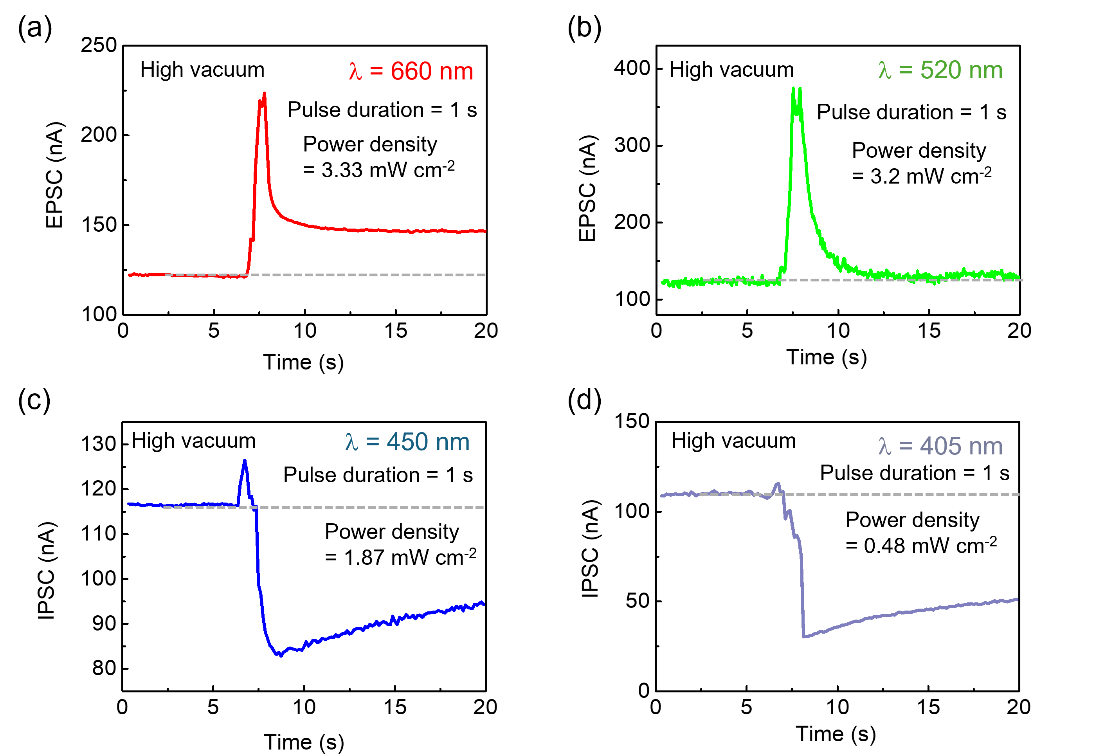


Figure S3. Postsynaptic current (PSC) responses measured under high-vacuum conditions to (a) 660, (b) 520, (c) 450, and (d) 405 nm optical pulses (1 s), exhibiting wavelength-dependent programming behavior consistent with ambient measurements.

Control experiments were performed under high-vacuum conditions (≈ 5 × 10^-6^ Torr) to evaluate the influence of environmental effects. As shown in Figure S3, the device exhibits behavior consistent with that measured under ambient conditions: illumination at 660 nm and 520 nm induces high-conductance programming, whereas 450 nm and 405 nm illumination results in low-conductance programming. These results indicate that the device operation is largely insensitive to gas adsorption or desorption. This environmental robustness is attributed to the clean van der Waals surface of the MoS_2_/CIPS heterostructure, where the atomically sharp and dangling-bond-free interface minimizes adsorption sites and suppresses environment-induced charge modulation.


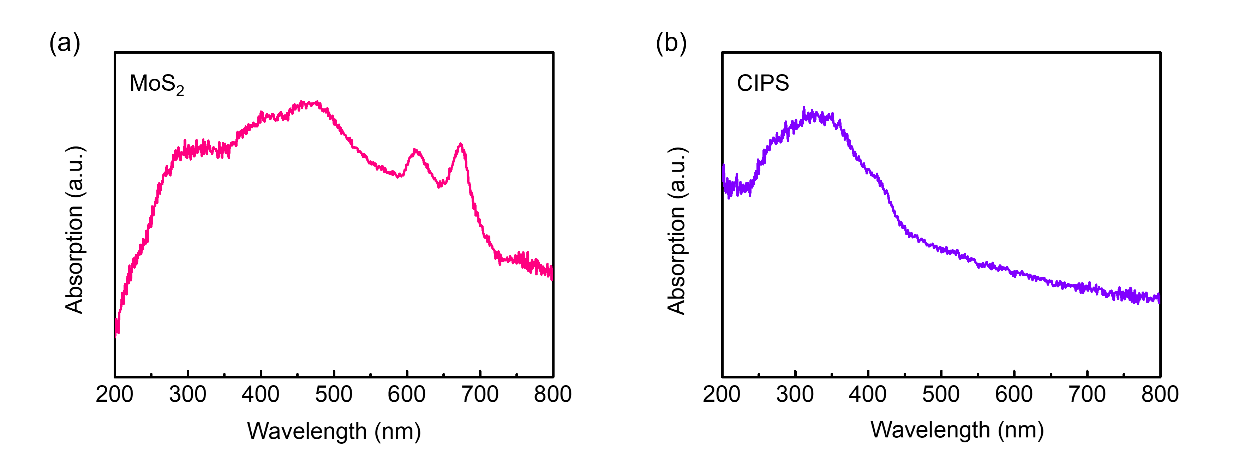


Figure S4. UV−vis absorption spectra of (a) MoS_2_ and (b) CIPS.

The optical absorption spectra of MoS_2_ and CIPS were measured in the wavelength range of 200–800 nm (Figure S4). MoS_2_ exhibits characteristic A and B exciton absorption peaks at approximately 660 nm and 610 nm (Figure S4a), consistent with high-quality few-layer MoS_2_.^[30,31]^ The strong absorption near 660 nm corresponds to the excitation wavelength used in optical synaptic experiments.

CIPS shows a broad absorption band spanning ~250–450 nm (Figure S4b), consistent with previous reports.^[32,33]^ The absorption at 450 nm corresponds to the blue-light excitation used in the experiments.

These absorption features indicate that MoS_2_ efficiently generates photocarriers under visible and near-UV illumination, whereas CIPS predominantly generates photocarriers under near-UV illumination, providing a basis for the wavelength-dependent optoelectronic responses observed in MoS_2_/CIPS heterostructure devices.


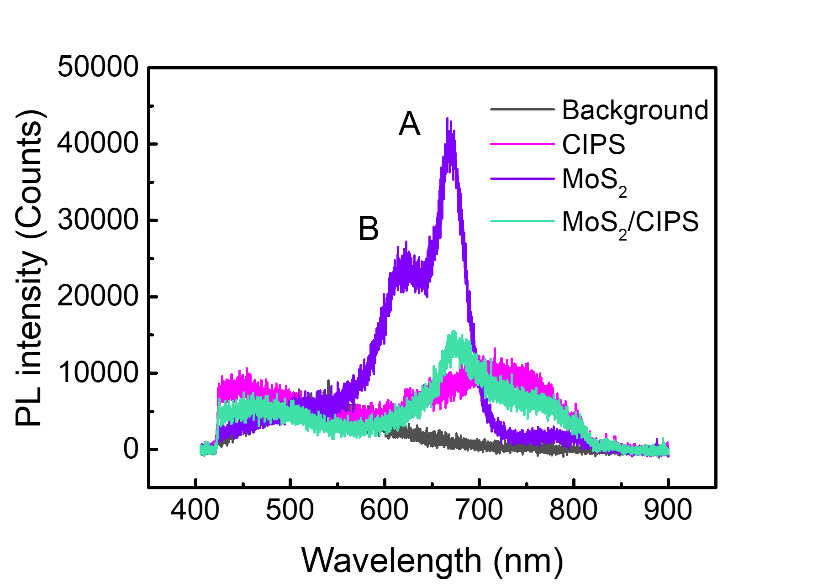


Figure S5. The photoluminescence emission spectra of MoS_2_, CIPS and MoS_2_/CIPS heterostructure with an excitation of the 405 nm laser.

Figure S5 shows the photoluminescence (PL) spectra of MoS_2_, CIPS, and the MoS_2_/CIPS heterostructure. Pristine MoS_2_ exhibits two prominent emission peaks corresponding to the A and B excitons at approximately 670 nm (≈ 1.85 eV) and 619 nm (≈ 2.0 eV), consistent with previous reports.^[34,35]^ Pristine CIPS displays PL emission centered around 450 nm (≈ 2.76 eV), corresponding to its bandgap.^[36]^ A sub-bandgap emission at 715 nm (≈ 1.73 eV) is also observed in bare CIPS, attributed to defect states within bandgap.^[37]^

Upon formation of the MoS_2_/CIPS heterostructure, the PL intensity of MoS_2_ is significantly quenched, indicating a reduced radiative recombination probability. The PL signal of CIPS shows a moderate decrease. This PL quenching behavior suggests efficient interfacial charge transfer and redistribution across the MoS_2_/CIPS van der Waals interface..^[38,39]^

**
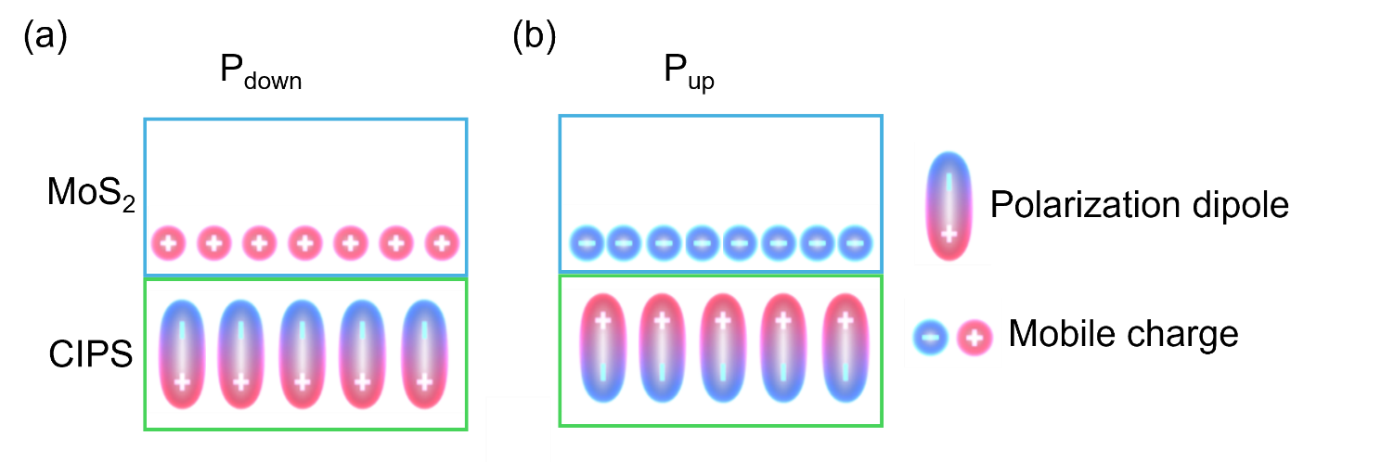
**

Figure S6. Illustration of the polarization state in the optical-FeFET with MoS_2_/CIPS heterostructure in dark. (a) CIPS is switched to the P_down_ state. (b) CIPS is switched to the P_up_ state.

When CIPS is polarized to the P_down_ state (Figure S6a), positive mobile charges accumulate at the MoS_2_/CIPS interface. In contrast, when CIPS is polarized to the P_up_ state (Figure S6b), negative mobile charges accumulate at the interface.

**
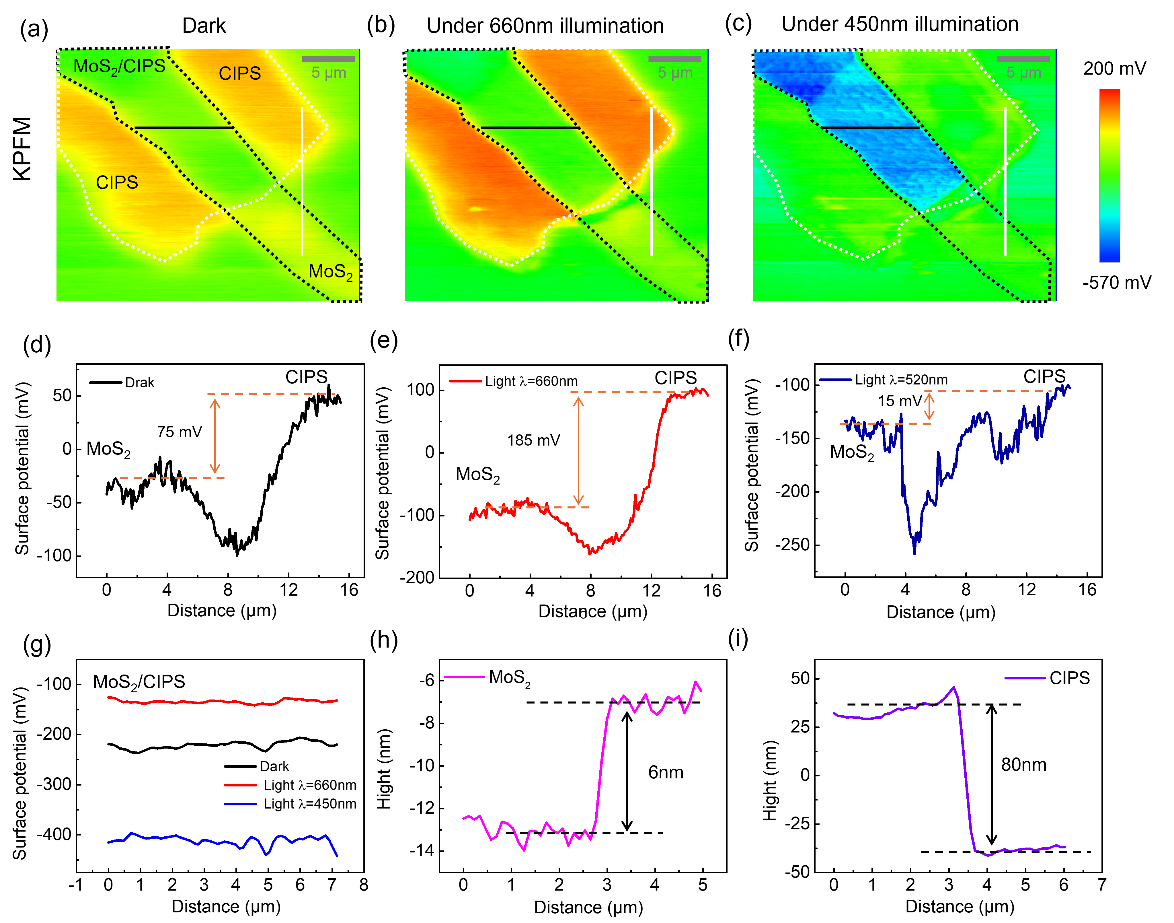
**

Figure S7. (a)–(c) KPFM image of MoS_2_, CIPS and MoS_2_/CIPS heterostructure (a) in dark, (b) under 660 nm illumination and (c) under 450 nm illumination. (d)–(f) Surface potential difference between MoS_2_ and CIPS (d) in dark, (e) under 660 nm illumination and (f) under 450 nm illumination. (g) Surface potential change of MoS_2_ with underlaying CIPS when in dark, under 660 nm or 450 nm illumination. Thickness of (h) MoS_2_ and (i) CIPS layers.

The surface potential distribution of the MoS_2_/CIPS heterojunction was measured using Kelvin probe force microscope (KPFM) under dark, 660 nm and 450 nm light illumination. The band alignment and the built-in field at interface under dark and under illumination is investigated. The KPFM surface potential mapping under dark, 660 nm and 450 nm light illumination is displayed in Figures S7a–c, respectively. Under dark, the potential distribution along the white solid line in Figure S7a was plotted in Figure S7d. The contact potential difference (*V*_CPD_) between the tip of AFM and the sample is defined as:

$$V_{CPD1}=\frac{W_{tip}-W_{1}}{e}= -30 mV$$

$$V_{CPD2}=\frac{W_{tip}-W_{2}}{e}= 45 mV$$

where the *W*_tip_, *W*_1_ and *W*_2_ are the work functions of the AFM tip, MoS_2_, and CIPS, respectively. The subscripts 1 and 2 represent MoS_2_ and CIPS, respectively, in the following analyses of energy band. The potential difference is calculated as:

$$V_{D}= V_{CPD2}- V_{CPD1}=75 mV$$

Then, the work function difference *W*_1_ – *W*_2_ = 75 meV can be obtained (Figure S8a). As a result, upon contact between MoS_2_ and CIPS, a built-in electric field pointing from CIPS to MoS_2_ is established, with the energy bands of CIPS bending upward and those of MoS_2_ bending downward at the interface (Figure S8b). This behavior is consistent with previously reported studies.^[40,41]^”

Under 660 nm illumination, the surface potential of MoS_2_ decreases, whereas that of CIPS increases, resulting in an enhanced potential difference of ~185 mV between CIPS and MoS_2_ (Figures S7b and e). Under this excitation, photocarriers are predominantly generated in MoS_2_ and are efficiently separated by the built-in electric field at the heterojunction, with photoexcited electrons preferentially transferring from MoS_2_ to CIPS (Figure 2d(i)). The 660 nm illumination corresponds to a photon energy of 1.88 eV, which exceeds the bandgap of MoS_2_ (~1.2 eV) and is sufficient for photoexcited electrons to overcome the conduction band offset between MoS_2_ and CIPS (~0.3 eV; Figure S8), while remaining insufficient to overcome the much larger valence band offset (~1.5 eV; Figure S8). Consequently, interfacial carrier transfer under 660 nm illumination is dominated by hot electrons, leading to the observed enhancement in the surface potential difference. The corresponding band diagram is shown in Figure S9a.

Under 450 nm illumination, the surface potential of both MoS_2_ and CIPS decrease, and the potential difference between them is reduced to ~15 mV (Figures S7c and f). Under this excitation, a large number of photocarriers are generated in both MoS_2_ and CIPS. In CIPS, photogenerated holes drift toward the MoS_2_/CIPS interface and the MoS_2_ layer, whereas photogenerated electrons are driven away from the interface due to the upward band bending of CIPS at the MoS_2_/CIPS junction. Owing to the significantly higher conductivity of MoS_2_ compared to CIPS, the depletion region of the heterojunction is primarily located within the CIPS layer, which further enhances the built-in field directing hole transport toward the interface. Consequently, the surface potentials of both MoS_2_ (away from the interface) and CIPS (near the interface) decrease, and the potential difference between the CIPS and the MoS_2_ is reduced. The corresponding band diagram is shown in Figure S9b.


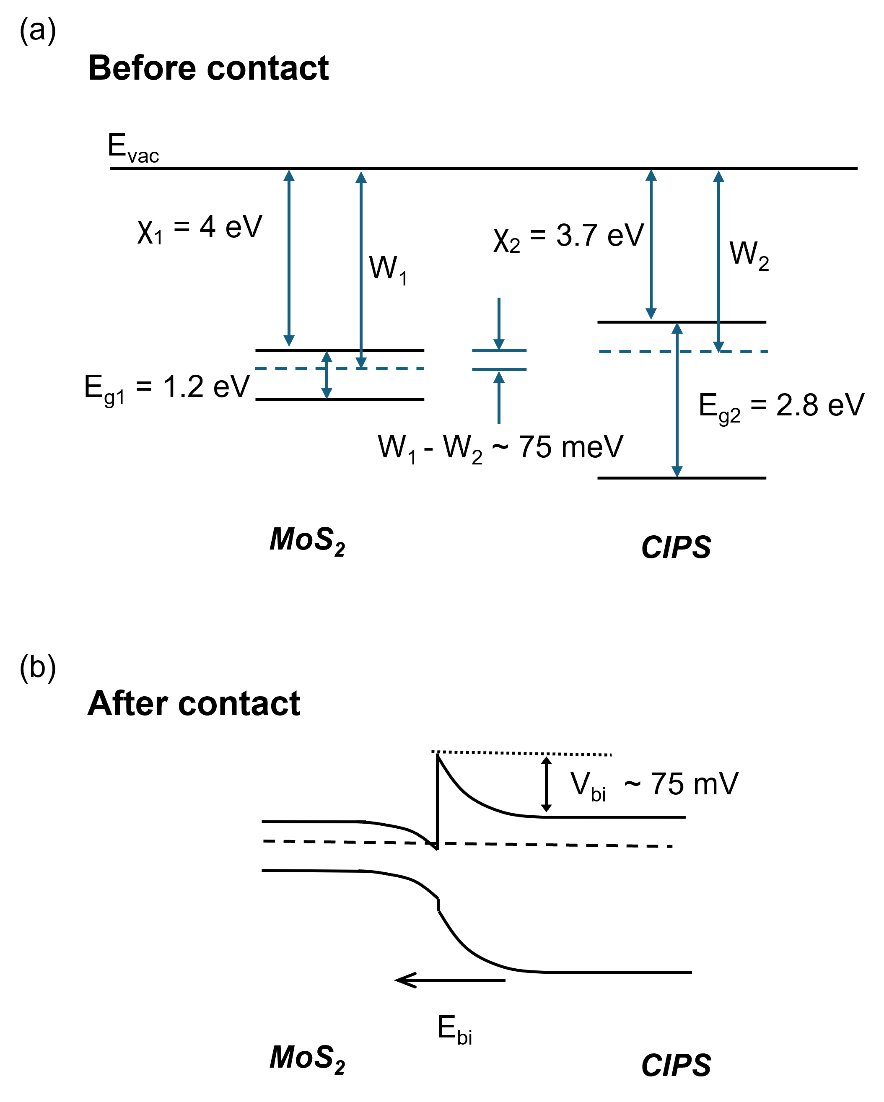


Figure S8. (a) The band diagram of MoS_2_ and CIPS before contact. (b) The band diagram of the contacted MoS_2_/CIPS heterostructure, a built-in electric field (E_bi_) is pointed from CIPS to MoS_2_.

As shown in Figure S8a, MoS_2_ has an electron affinity (X_1_) of ~4 eV and a bandgap (E_g1_) of ~1.2 eV, ^[19]^ while CIPS has an electron affinity (X_2_) of ~3.7 eV and a bandgap (E_g2_) of ~2.8 eV. ^[20]^ Due to the difference between work function (W_1_) of MoS_2_ and that of CIPS (W_2_), where (ΔW = W1 – W2) is approximately 75 meV (Figure S7), results in the formation of a built-in potential (V_bi_ ~ 75mV) at the MoS_2_/CIPS interface and the establishment of a built-in electric field (E_bi_) directed from CIPS to MoS_2_ (Figure S8b).^[21,22]^


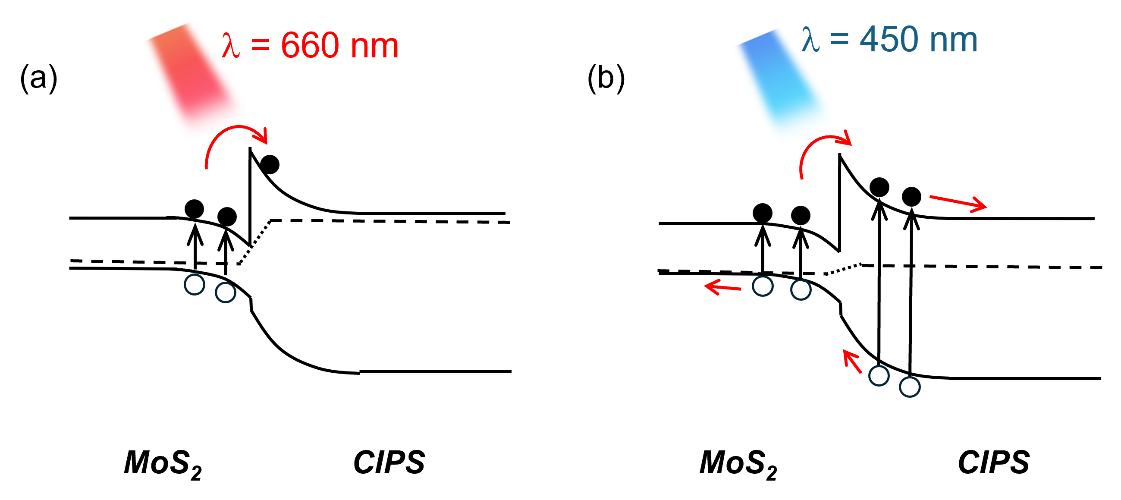


Figure S9. Schematic illustration of carrier transfer in the MoS_2_/CIPS heterojunction under (a) 660 nm and (b) 450 nm light illumination.

Figure S9 illustrates the band diagrams and carrier transfer processes in the MoS_2_/CIPS heterojunction under 660 nm (Figure S9a) and 450 nm (Figure S9b) illumination. As discussed in Figure S7, under 660 nm illumination, the transferred electrons accumulate at the MoS_2_/CIPS interface and the top surface of CIPS (Figure S9a), where they effectively screen the positive bound polarization charges of CIPS, thereby stabilizing the upward polarization state. In contrast, under 450 nm illumination, the accumulation of holes near the interface contributes to electrostatic screening of the opposite bound polarization charges, making the downward polarization energetically favorable in this regime.


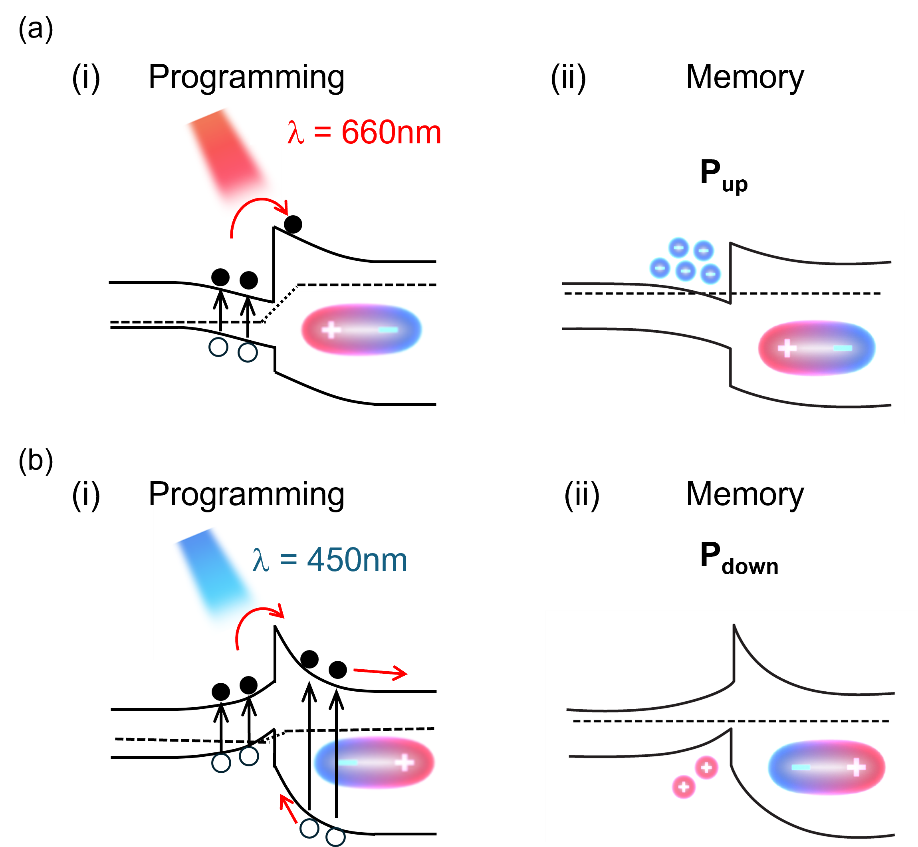


Figure S10. Illustration of the band diagrams of the optical FeFET with an MoS_2_/CIPS heterostructure: (a) Programming with longer-wavelength light (e.g., 660 nm) when the polarization is initially upward. (i) During 660 nm light programming. (ii) Memory state after 660 nm light programming. (b) Programming with shorter-wavelength light (e.g., 450 nm) when the polarization is initially downward. (i) During 450 nm light programming. (ii) Memory state after 450 nm light programming.

When the device is programmed with longer-wavelength light (e.g., 660 nm) and the polarization of CIPS is initially upward (Figure S10a(i)), the photogenerated carriers in MoS_2_ are efficiently separated by the built-in electric field at the heterojunction, with photoexcited electrons preferentially transferring from MoS_2_ to CIPS. The transferred electrons accumulate at the MoS_2_/CIPS interface and the top surface of CIPS, where they effectively screen the positive polarization charges of CIPS. As a result, the upward polarization is stabilized through electrostatic interaction and thus maintained (Figure S10a(ii)). The case of programming with 660 nm light under an initially downward polarization is shown in Figure 2d.

In contrast, when the device is programmed with shorter-wavelength light (e.g., 450 nm) and the polarization of CIPS is initially downward (Figure S10b(i)), above-bandgap illumination generates photoexcited electron–hole pairs in CIPS. In CIPS, photogenerated holes drift toward the MoS_2_/CIPS interface and the MoS_2_ layer, whereas photogenerated electrons are driven away from the interface due to the upward band bending of CIPS at the MoS_2_/CIPS junction. The accumulation of holes near the interface contributes to electrostatic screening of the opposite bound polarization charges, which makes the downward polarization energetically favorable in MoS_2_/CIPS heterostructure. Consequently, the downward polarization is preserved (Figure S5b(ii)). The case of programming with 450 nm light under an initially upward polarization is shown in Figure 2e.

**
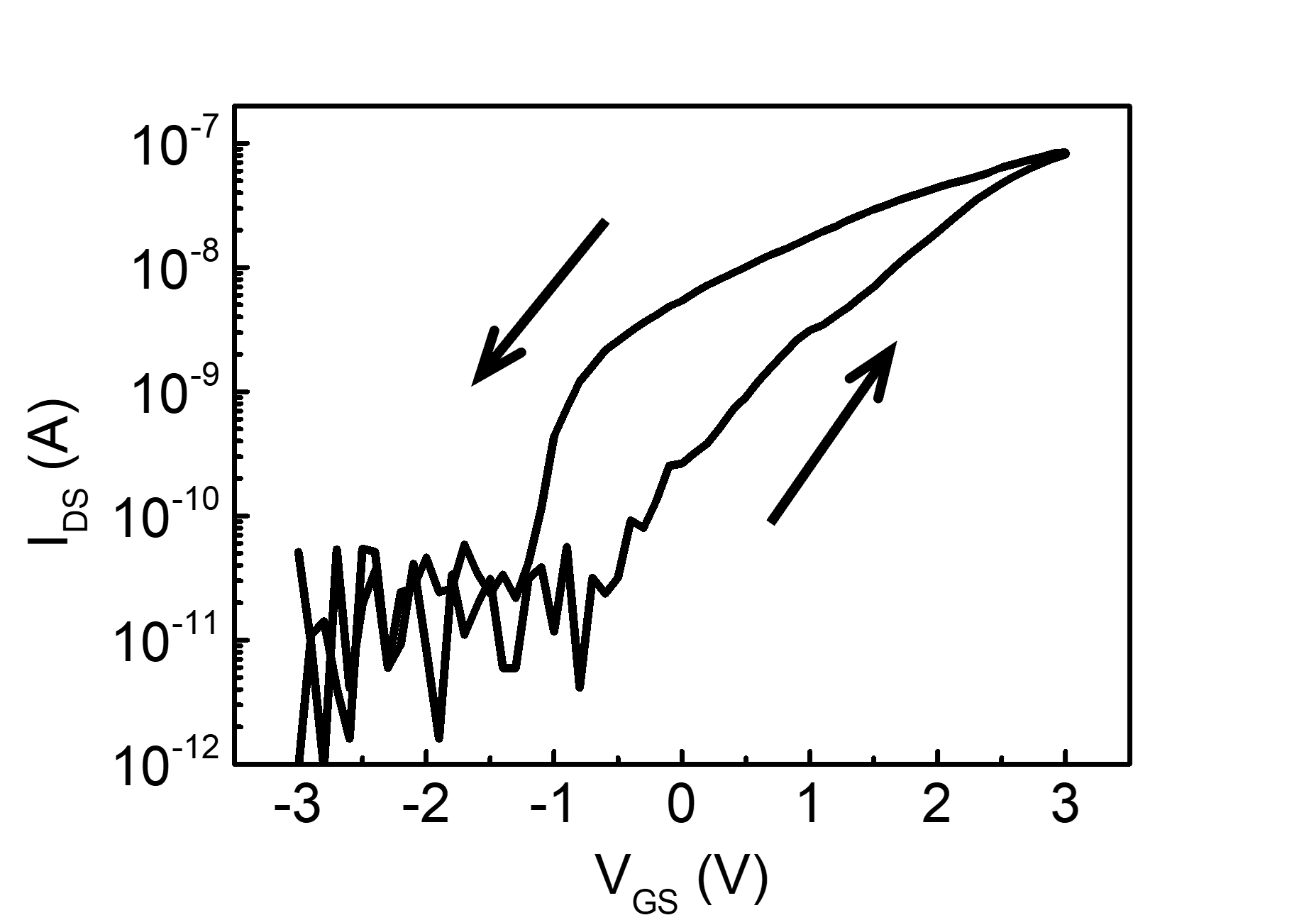
**

Figure S11. Transfer curve of the optical-FeFET.

Figure S11 presents the *I_DS_*−*V_GS_* characteristics of the optical-FeFET device. During bidirectional scanning, the gate voltage (*V_GS_*) varied from 3 V to +3 V and then returned to −3 V, while the drain voltage (*V_DS_*) was maintained at 0.5 V. The device exhibits a distinct anticlockwise hysteresis, characteristic of ferroelectric polarization switching, thereby confirming the effective ferroelectric behavior of the optical-FeFET.

**
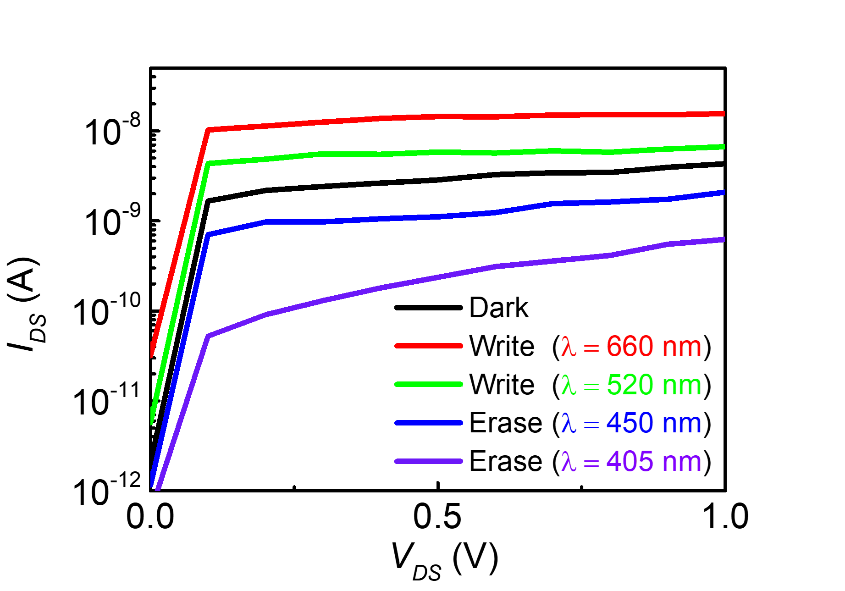
**

Figure S12. *I_DS_–V_DS_* characteristics of the optical-FeFET, demonstrating conductance modulation under four different wavelengths: 660, 520, 450, and 405 nm.

As shown in Figure S12, the *I_DS_–V_DS_* characteristics were measured in the dark and after a 1 s illumination with 660, 520, 450, and 405 nm light. Illumination with 660 nm and 520 nm light induces an increase in conductance, whereas illumination with 450 nm and 405 nm light induces a decrease in conductance. Compared to 660 nm, illumination at 520 nm produces weaker conductance modulation and a lower channel current, owing to the interplay of two mechanisms as discussed in Figure 2 of the main text. Furthermore, illumination at 405 nm generates stronger conductance modulation and a lower channel current compared to 450 nm, resulting from the larger number of photo-induced carriers and the accumulation of positive charges at the interface, which promotes additional dipole switching in the downward direction (see discussion in Figure 2 of the main text).

**
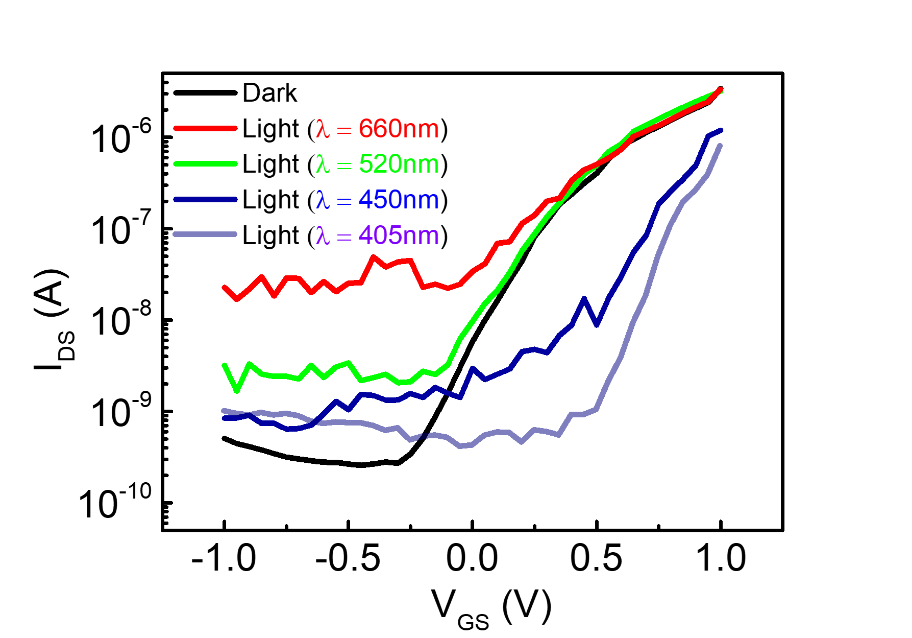
**

Figure S13. *I_DS_–V_GS_* characteristics of the optical-FeFET under exposure to four different wavelengths: 660, 520, 450, and 405 nm.

As shown in the *I_DS_–V_GS_* curves (Figure S13), the device exhibits positive photocurrent under 660 nm and 520 nm exposure, whereas negative photocurrent is observed under 450 nm and 405 nm exposure. These results are consistent with the polarization switching directions, where 660 nm and 520 nm light drive the polarization upward (yielding higher conductance), while 450 nm and 405 nm light drive it downward (yielding lower conductance).

**
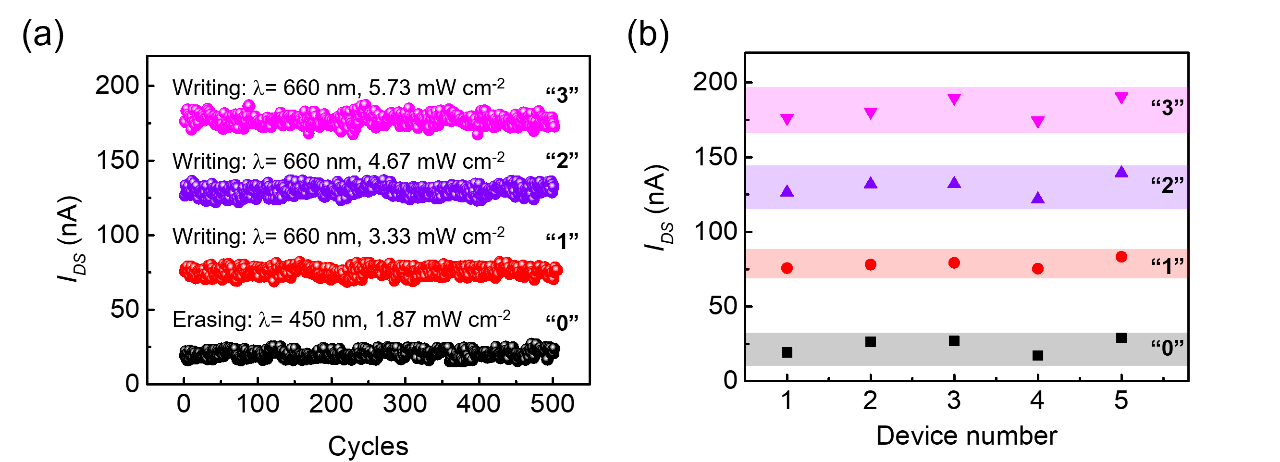
**

Figure S14. (a) Multilevel memory states over 500 programming cycles on the same device. (b) Multilevel memory states for 5 devices.

For multilevel optical memory characterization, repeated optical writing and erasing operations were conducted over 500 programming cycles on the same device (Figure S14a). In addition, five devices were evaluated under identical conditions for single-cycle measurements (Figure S14b). Notably, the conductance separation between adjacent memory levels is larger than the corresponding statistical variation, resulting in non-overlapping conductance distributions. These results demonstrate that all intermediate states are distinguishable and reliable for multilevel optical memory operation.


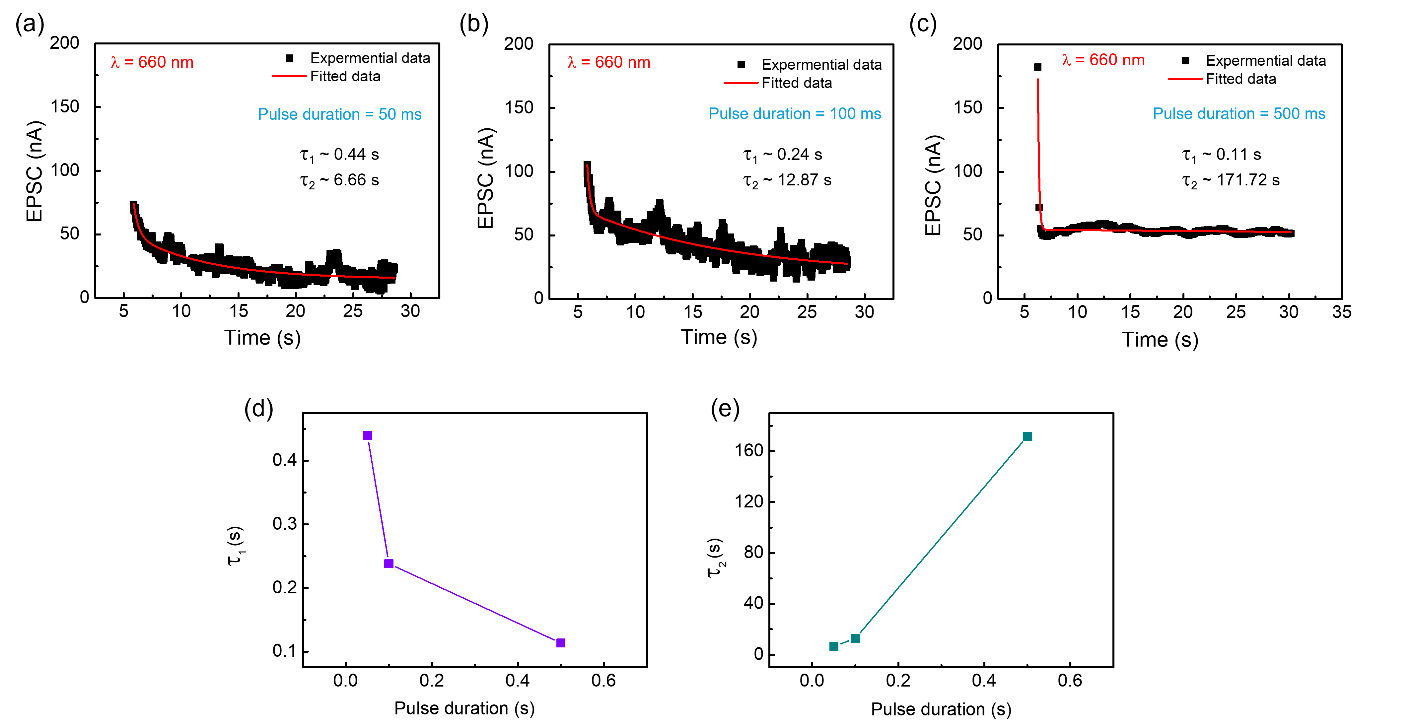


Figure S15. (a)–(c) Time-resolved photocurrent and fitting after turning off 660 nm light with light duration of (a) 50 ms, (b) 100 ms and (c) 500 ms. Extracted time constant (d) τ_1_ and (e) τ_2_ as function of 660 nm light pulse duration.

To further elucidate the transition from short-term memory (STM) to long-term memory (LTM), time-resolved photocurrent measurements were performed using short optical pulses, followed by a systematic analysis of the post-illumination relaxation dynamics. The temporal evolution of the photocurrent after optical excitation was fitted with a bi-exponential model to extract characteristic relaxation time constants associated with different carrier relaxation processes.

Time-resolved photocurrent traces under 660 nm illumination were recorded with pulse durations of 50 ms, 100 ms, and 500 ms (Figures S15a–c), while the corresponding relaxation behaviors under 450 nm illumination were measured with pulse durations of 100 ms, 500 ms, and 1 s (Figures S16a–c). The relaxation dynamics were fitted using the bi-exponential function:

$$EPSC(t)/IPSC(t) =A_{1}\exp\left( -{\frac{t}{\tau}}_{1} \right) + A_{2}\exp\left( -{\frac{t}{\tau}}_{2} \right)+I_{0}$$

where *A_1_* and *A_2_* represent the amplitudes of the fast and slow relaxation components, respectively, τ_1_ and τ_2_ are the corresponding relaxation time constants, and *I_0_* denotes the baseline current.

Across both excitation wavelengths, the photocurrent relaxation exhibits two characteristic time scales, indicating multiple carrier relaxation and redistribution pathways at the MoS₂/CIPS interface. The fast component (τ_1_) is associated with rapid photo-carrier redistribution, interfacial charge transfer, and initial screening of the ferroelectric polarization, whereas the slow component (τ_2_) reflects long-lived carrier relaxation governed by carrier–polarization coupling and interfacial stabilization at the ferroelectric interface.

As summarized in Figure S15d, under 660 nm illumination both τ_1_ and τ_2_ show a dependence on the optical pulse duration. While τ_1_ varies only weakly, τ_2_ increases markedly with increasing pulse duration. In particular, when the pulse duration is increased from 100 ms to 500 ms, τ_2_ increases from 12.87 s to 171.72 s, indicating a transition from a short-lived relaxation regime to a long-lived, stabilized conductance state. This pronounced extension of the slow relaxation time provides kinetic evidence for the transition from STM-like to LTM-like behavior.


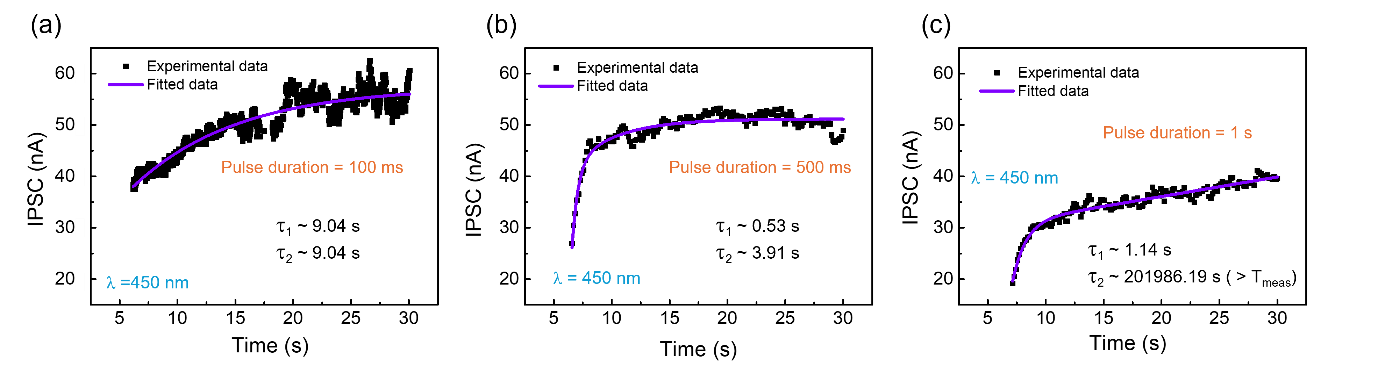


Figure S16. (a)–(c) Time-resolved photocurrent and fitting after turning off 450 nm light with light duration of (a)100 ms, (b) 500 ms and (c) 1 s.

Under 450 nm optical stimulation (Figure S16), the relaxation dynamics exhibit a different evolution compared with the 660 nm case, reflecting a distinct carrier stabilization process. When the pulse duration is limited to 100 ms (Figure S16a), the post-illumination photocurrent decay is described by a single-exponential behavior, with τ_1_ and τ_2_ converging to an identical value of approximately 9.04 s. This convergence indicates that relaxation is governed by a single dominant carrier relaxation channel, characteristic of volatile, STM-like behavior.

As the pulse duration is increased to 500 ms (Figure S16b), the relaxation dynamics evolve into a bi-exponential form, with a fast component (τ_1_ ≈ 0.53 s) and a slower component (τ_2_ ≈ 3.91 s). This emergence of two-time constants suggests the coexistence of fast charge redistribution and weakly stabilized carrier–polarization charge coupling at the MoS_2_/CIPS interface. However, both relaxation processes remain within the short time-scale regime, indicating that the interaction between photocarriers and ferroelectric polarization is insufficient to establish a long-lived, nonvolatile conductance state, and the device response remains predominantly STM-like.

When the pulse duration reaches 1 s (Figure S16c), the extracted slow relaxation time constant (τ_2_) increases dramatically to a value far exceeding the experimental observation window (τ_2_ ≫ measurement time scale), while the fast component remains on the order of seconds (τ_1_ ≈ 1.14 s). This apparent divergence of τ_2_ reflects the formation of stabilized interfacial charge configuration mediated by ferroelectric polarization under prolonged excitation.

In conclusion, the transition from short-term memory (STM) to long-term memory (LTM) in the MoS_2_/CIPS heterostructure is governed by a critical switching condition defined by the optical stimulation parameters. Specifically, when the optical pulse duration exceeds a critical value, the relaxation dynamics of the device undergo a change, characterized by a sudden and substantial increase in the slow relaxation time constant (τ_2_).

The following analysis is combined with carrier relaxation dynamics.

Under 660 nm optical excitation (Figures S15), the extracted τ_1_ remains on the order of seconds with only weak dependence on the pulse duration (Figures S15d), suggesting that fast photo-carrier interfacial redistribution processes are governed primarily by intrinsic material and interface properties (e.g., the built-in electric field at MoS_2_/CIPS interface). In contrast, the slow relaxation component τ_2_ exhibits a pronounced increase with increasing pulse duration (Figures S15e), indicating that prolonged excitation leads to enhanced carrier accumulation and stronger coupling between photogenerated carriers and the ferroelectric polarization in CIPS. This behavior implies a gradual evolution from transient carrier redistribution to a more stabilized interfacial charge configuration mediated by ferroelectric polarization.

Under 450 nm excitation (Figures S16), the relaxation dynamics show a different evolution. For short pulse durations (100 ms) (Figures S16a), the decay behavior is described by a single-exponential response, with τ_1_ and τ_2_ converging to a similar value (~9.04 s), indicating that carrier relaxation is dominated by a single effective pathway. With increasing pulse duration (Figures S16b), relaxation evolves into a bi-exponential form, revealing the emergence of a fast component (τ_1_) associated with rapid photo-carrier redistribution and a slower component (τ_2_) related to delayed carrier relaxation influenced by ferroelectric polarization. For the longest pulse duration (1 s) (Figures S16c), the slow relaxation component becomes significantly extended beyond the experimental observation window, suggesting the formation of a stationary interfacial carrier population stabilized by cumulative ferroelectric polarization.

Overall, the bi-exponential fitting of time-resolved photocurrent transients provides kinetic evidence for wavelength- and excitation-dependent carrier relaxation pathways in the MoS_2_/CIPS heterostructure. The extracted time constants reveal how photogenerated carriers undergo rapid redistribution followed by slower polarization-coupled relaxation processes, highlighting the critical role of built-in electric field at interface and ferroelectric polarization in modulating photo-carrier dynamics at the heterointerface.


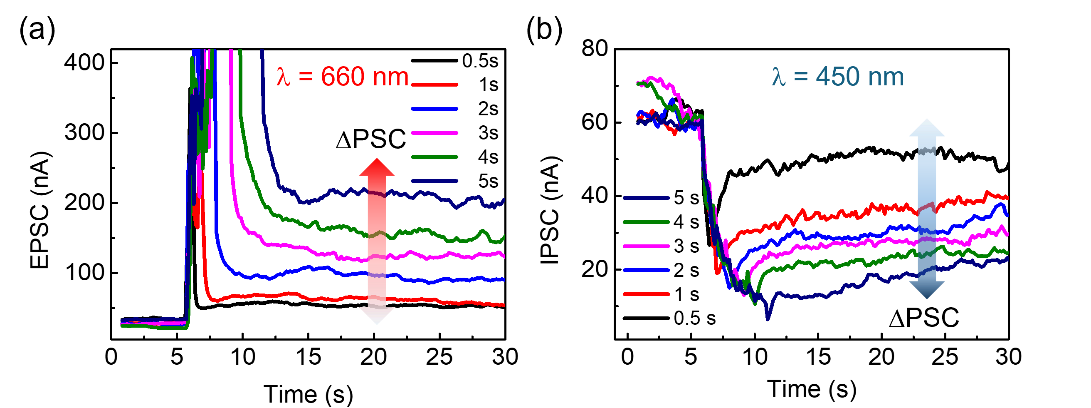


Figure S17. Light duration-dependent responses of the optical-FeFET. (a) EPSC response when wavelength is 660 nm for different light pulse durations (0.5, 1, 2, 3, 4, and 5 s). The pulse power intensity is 2.1 mW cm^-2^. (b) IPSC response when wavelength is 450 nm with different light pulse durations (0.5, 1, 2, 3, 4, and 5 s). The pulse power intensity is 0.85 mW cm^-2^.

A single optical pulse with varying durations (0.5, 1, 2, 3, 4, and 5 s) was applied to investigate the relationship between stimulus duration and synaptic strength. As shown in Figures S17a and S17b, the EPSC/IPSC amplitude change increases monotonically with increasing pulse duration. This behavior is attributed to enhanced photocarrier generation and stronger polarization switching under prolonged illumination.


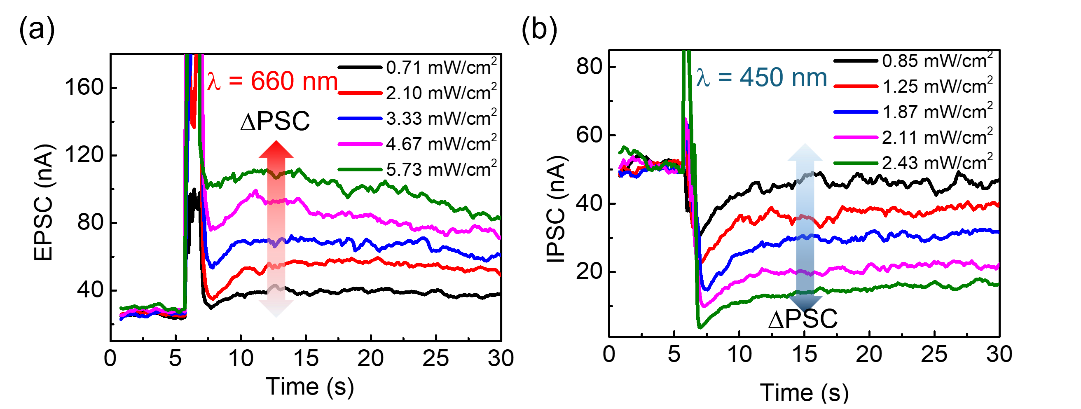


Figure S18. Light intensity-dependent responses of the optical-FeFET. (a) EPSC response when wavelength is 660 nm with different light pulse intensities (0.71, 2.1, 3.33, 4.67, and 5.73 mW cm^-2^). Pulse duration is 1 s. (b) IPSC response when wavelength is 450 nm with different light pulse intensities (0.85, 1.25, 1.87, 2.11, and 2.43 mW cm^-2^). Pulse duration is 1 s.

To investigate the relationship between stimulus intensity and synaptic strength, the device was subjected to single optical pulses with varying power densities (0.71, 2.1, 3.33, 4.67, and 5.73 mW cm^-2^ for 660 nm light, 0.85, 1.25, 1.87, 2.11, and 2.43 mW cm^-2^ for 450 nm light). As shown in Figures S18a and S18b, the EPSC/IPSC amplitude change increases with increasing incident light intensity.

**
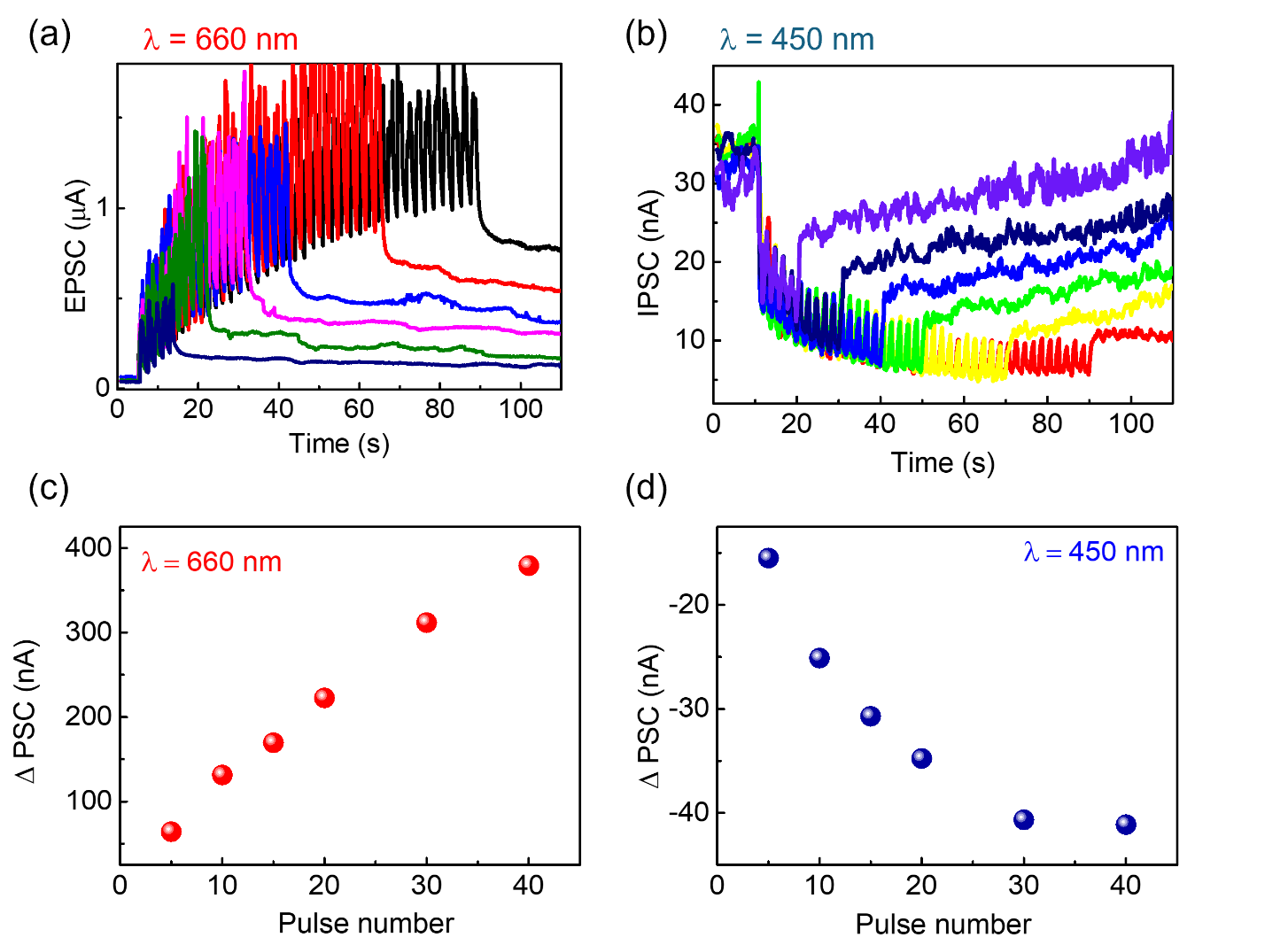
**

Figure S19. Spike-number-dependent plasticity (SNDP) of the optical-FeFET. (a) EPSC response when wavelength is 660 nm with different light pulse numbers (5, 10, 15, 20, 30, and 40). Pulse duration is 1 s. (b) IPSC response when wavelength is 450 nm with different light pulse numbers (5, 10, 15, 20, 30, and 40). Pulse duration is 1 s. (c), (d) Corresponding changes in postsynaptic current (ΔPSC, recorded 10 s after light irradiation) extracted from (a) and (b), respectively.

The spike-number-dependent plasticity (SNDP) of the device is summarized in Figures S19a and S19b, with the corresponding changes in postsynaptic current (ΔPSC, measured 10 s after light irradiation) shown in Figures S19c and S19d. Both EPSC and IPSC amplitudes increase monotonically with the number of pulses (5, 10, 15, 20, 30, and 40) under 660 nm and 450 nm illumination, respectively, accompanied by a transition from STP/STD to LTP/LTD. Specifically, ΔPSC increases from 64.27 to 379.3 nA under 660 nm (Figure S19c) and decreases from -15.49 to -41.13 nA under 450 nm as the pulse number increases from 5 to 40 (Figure S19d).

**
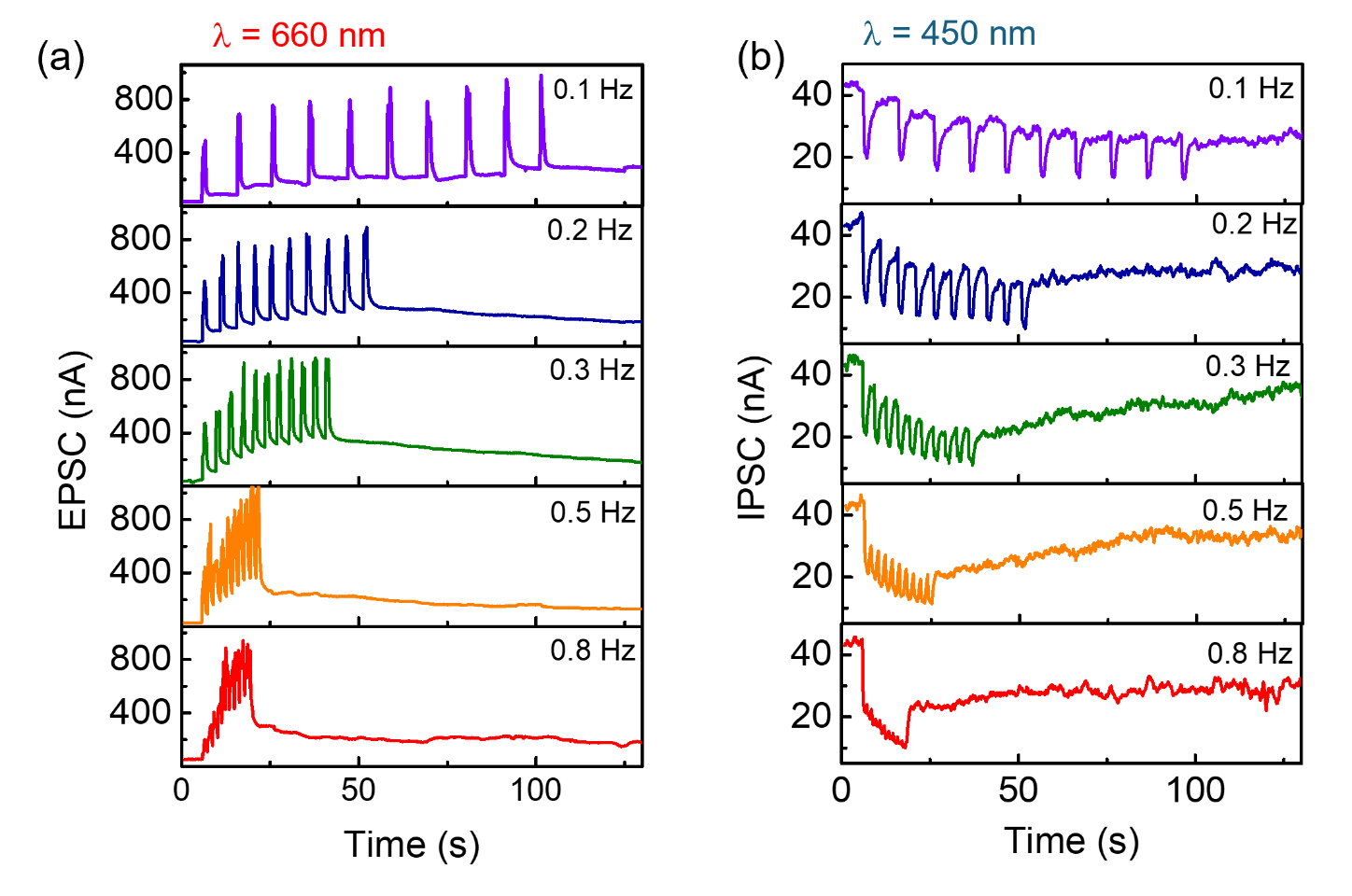
**

Figure S20. Spike-frequency-dependent plasticity (SFDP) of the optical-FeFET. (a) EPSC response when wavelength is 660 nm with different light pulse frequencies (0.2, 0.3, 0.5, and 0.8 Hz). Pulse duration is 1 s. (b) IPSC response when wavelength is 450 nm with different light pulse frequencies (0.2, 0.3, 0.5, and 0.8 Hz). Pulse duration is 1 s.

The spike-frequency-dependent plasticity (SFDP) of the device is summarized in Figures S20a and S20b. Under 660 nm and 450 nm illumination, both EPSC and IPSC amplitudes increase monotonically with the light pulse frequency (0.2, 0.3, 0.5, and 0.8 Hz), accompanied by a transition from STP/STD to LTP/LTD.

**
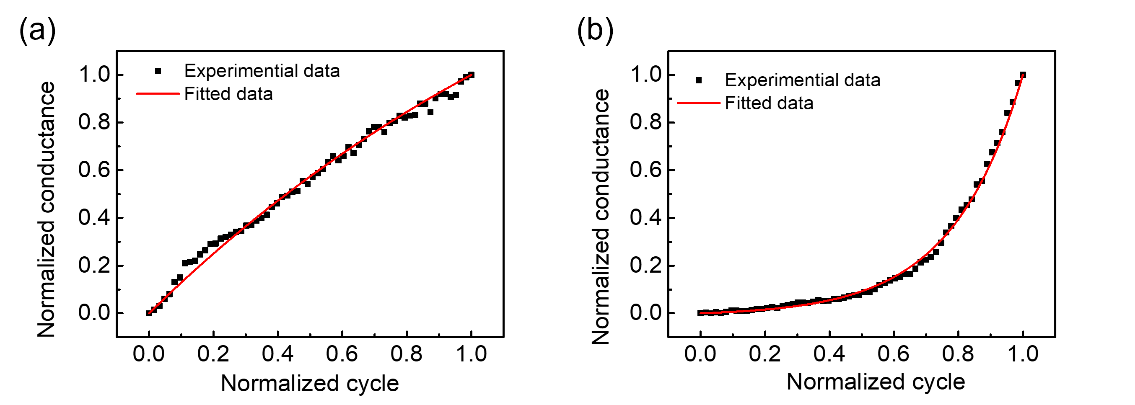
**

Figure S21. Nonlinearity analysis of LTP and LTD in the optical-FeFET synaptic device. (a) Nonlinearity fitting of LTP. (b) Nonlinearity fitting of LTD.

The synaptic weights used in the neural network simulations were directly extracted from the experimentally measured potentiation and depression characteristics (Figure S21). The conductance evolution under optical stimuli was fitted to obtain key parameters, including the dynamic range and nonlinearity factors of potentiation and depression.

The dynamic range is defined as *G_max_*/*G_min_*, where *G_max_* and *G_min_* correspond to the maximum and minimum conductance, respectively. The dynamic range of the device reaches approximately 24.2.

The nonlinearity (NL) of the LTP/LTD curves was calculated using the following equations:

$$G_{LTP}=B\left( 1-e^{\left( -\frac{P}{A_{P}} \right)} \right)+G_{min}$$

$$G_{LTD}=-B\left( 1-e^{\left( \frac{P-P_{max}}{A_{D}} \right)} \right)+G_{max}$$

$$B={(G}_{max}-G_{min})/(1-e^{\left( \frac{-P_{max}}{A_{P,D}} \right)})$$

*G_LTP_* and *G_LTD_* denote the conductance during long-term potentiation (LTP) and long-term depression (LTD), respectively, while *G_max_*, *G_min_*, and *P* correspond to the maximum conductance, minimum conductance, and the number of applied pulses. *P_max_* indicates the maximum pulse number. Parameters *A_P_* and *A_D_* are used to quantify the NL factors for potentiation and depression, respectively, and $B$serves as a fitting constant for conductance normalization. In this work, the *A* values are adopted following the established NeuroSim+ modeling framework.^[24]^ Based on these parameters, the corresponding NL factors can be obtained.

As shown in Figures S21a and b, the measured LTP and LTD characteristics were first normalized and then fitted using the analytical expressions described above. The fitting yields *A_P_* = 1.672 and *A_D_* = 0.196, resulting in extracted NL factors of 0.7 for LTP and 4.9 for LTD.

**
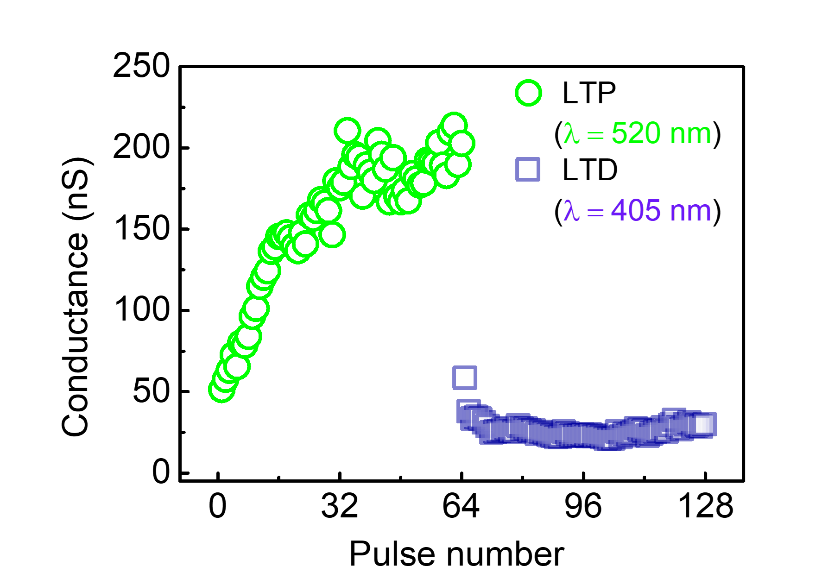
**

Figure S22. Long-term potentiation (LTP) at 520 nm and long-term depression (LTD) at 405 nm in an all-optical mode.

Long-term potentiation (520 nm) and depression (405 nm) are realized in all-optical mode using 64 pulses (1 s duration, 0.5 Hz frequency). The dynamic range, defined by the conductance ratio G_max_/G_min_, reaches approximately 5, confirming robust modulation of synaptic weights.


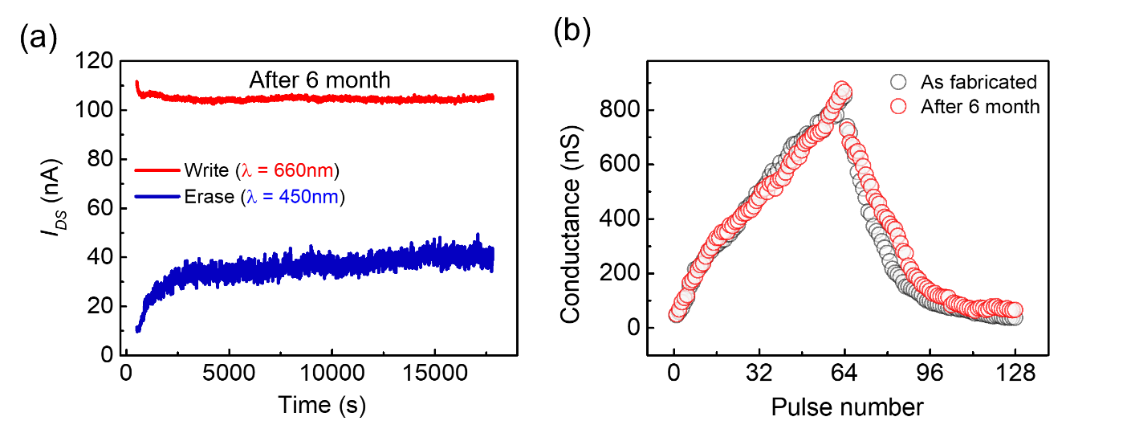


Figure S23. (a) Long-term retention (identical to Figure 3e) and (b) Light-induced synaptic plasticity (LTP/LTD) of the MoS_2_/CIPS device after ~6 months of ambient storage.

Table S4. Nonlinearity comparison of fabricated devices and the same device stored for 6 months.

| Device | LTP nonlinearity | LTD nonlinearity |
| --- | --- | --- |
| As fabricated | 0.7 | 4.9 |
| After 6 months | 0.31 | 3.55 |

The long-term stability of the MoS_2_/CIPS device was examined under ambient atmospheric conditions without encapsulation. After storage for approximately six months, the device exhibits negligible degradation in its electrical characteristics (Figure S23a). The light-induced synaptic plasticity is also largely preserved, with only a slight reduction in nonlinearity, as shown in Figure S23b and summarized in Table S4.


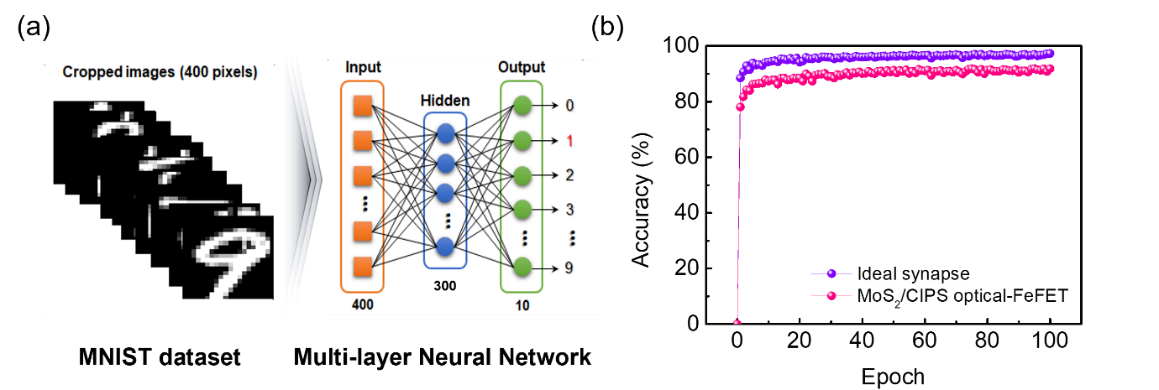


Figure S24. (a) Schematic of a three-layered ANN model with 300 neurons in hidden layer, for recognizing handwritten digit images based on the optical-FeFET. (b) Image recognition accuracy over 100 training epochs using the MNIST database.

Table S5. ANN simulation performance comparison between our device and recent reported all-optical synapses.

| Number of layers | Number of neurons in hidden layer | Number of training set | Training number per epoch | Epoch | Device/ideal Accuracy  (%) | Ref |
| --- | --- | --- | --- | --- | --- | --- |
| 3 | 300 | N.A. | N.A. | 40 | 95.33/98.00 | ^[18]^ |
| 3 | 300 | N.A. | N.A. | 30 | 92.2/N.A. | ^[16]^ |
| 3 | 300 | N.A. | N.A. | 40 | 90.8/98.1 | ^[5]^ |
| 3 | 100 | 60,000 | 1,000 | 20 | 86.82/N.A. | ^[6]^ |
| 3 | 64 | N.A. | N.A. | 1,000 | 94/N.A. | ^[12]^ |
| 3 | 300 | 60,000 | N.A. | 40 | 91/98 | ^[11]^ |
| 1 | N.A. | 10,000 | N.A. | 50 | 90.4/93.6 | ^[9]^ |
| 1 | N.A. | 60,000 | N.A. | 10,000 | 90/N.A. | ^[1]^ |
| 3 | 300 | 60,000 | 12,000 | 100 | 91.89/97.34 | This work |

To evaluate scalability, additional simulations were performed using a larger network configuration with 400 input neurons and 300 hidden neurons, and the number of training samples was increased to 12,000 per epoch. As shown in Figure S24, under this configuration, both ideal-device-based and experimentally extracted (real-device-based) models exhibited an approximately 1% improvement in recognition accuracy, indicating that the performance is largely independent of network size and can be extended to larger architectures.

The ANN simulation performance of our device was further compared with recently reported all-optical synapses, as summarized in Table S5. Most reported all-optical synapses employ 3-layer MLPs, while some use single-layer configurations without hidden layers. The number of training samples in our work is larger than that in some reports and comparable to others. Our device achieves an accuracy of 91.89%, which is higher than or comparable with those of recent all-optical synapses.


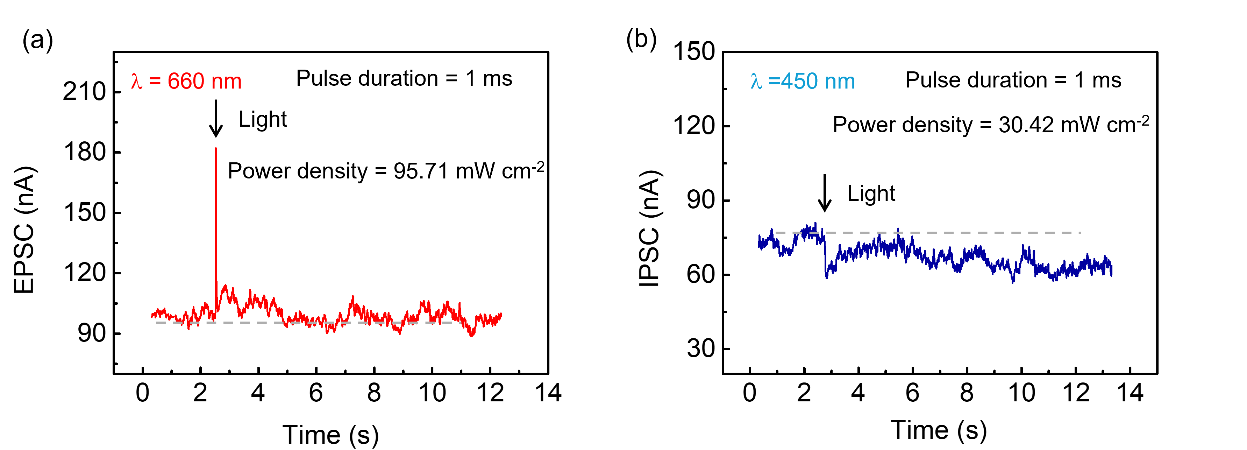


Figure S25. Conductance modulation under (a) 660 nm (95.71 mW cm^-2^) and (b) 450 nm (30.42 mW cm^-2^) illumination demonstrates optical switching with pulse widths down to 1 ms.

As shown in Figure S25, under increased optical power densities of 95.71 mW cm^-2^ at 660 nm and 30.42 mW cm^-2^ at 450 nm, the device exhibits optical switching with pulse widths down to 1 ms. This result indicates that the second scale switching time observed under low-power illumination is not a fundamental limitation but originates from the chosen operating regime. By increasing the optical power density, the device operation can be extended to the millisecond timescale.

Figure S26. Synaptic plasticity measured for devices with different MoS_2_ and CIPS thicknesses.


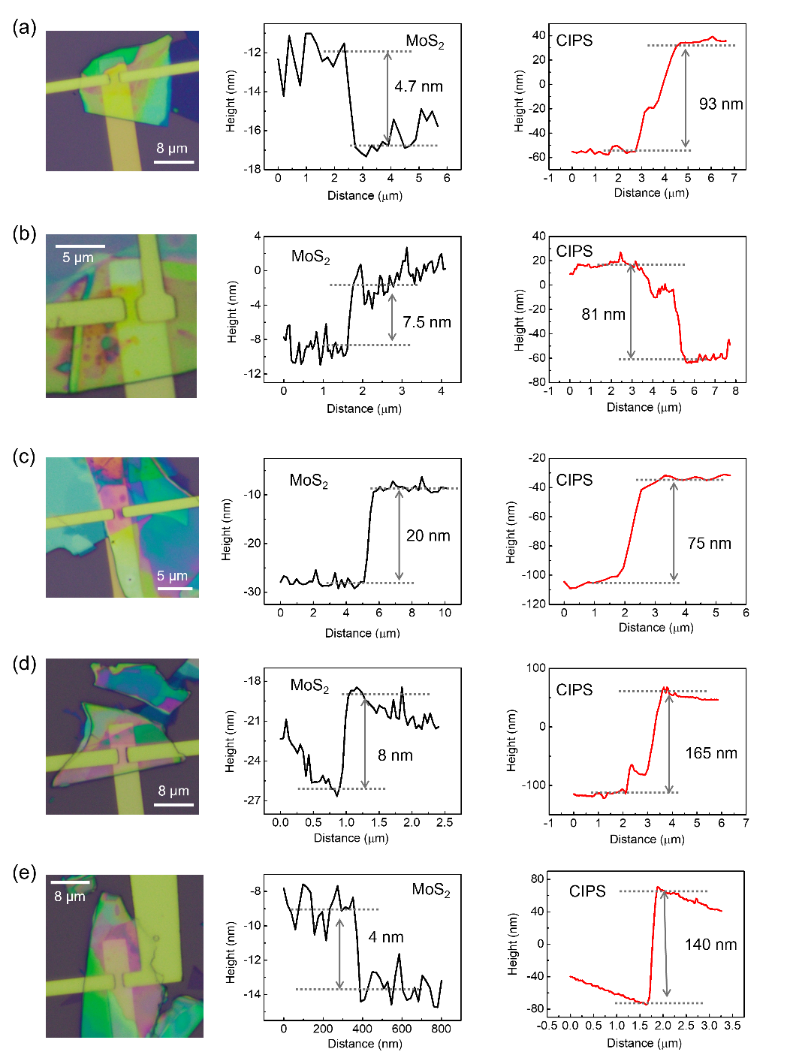


Figure S27. (a)–(e) Optical microscope images (left) and measured thicknesses of MoS_2_ (middle) and CIPS (right) for devices (a) #2, (b) #3, (c) #4, (d) #5, and (e) #6.

Table S6. Statistical analysis of synaptic plasticity and linearity across multiple devices.

| Device | CIPS thickness (nm) | MoS_2_ thickness (nm) | Dynamic range | LTP nonlinearity | LTD  nonlinearity |
| --- | --- | --- | --- | --- | --- |
| Device #1 | 79 | 5 | 24.2 | 0.7 | 4.9 |
| Device #2 | 93 | 4.7 | 33.8 | 0.94 | 3.43 |
| Device #3 | 81 | 7.5 | 23.4 | 1.31 | 5.75 |
| Device #4 | 75 | 20 | 31.3 | 0.13 | 5.11 |
| Device #5 | 165 | 8 | 9.3 | 1.85 | 3.81 |
| Device #6 | 140 | 4 | 14.5 | 0.33 | 3.48 |

To address concerns regarding device-to-device variability and thickness effects, we fabricated and characterized six MoS_2_/CIPS devices with intentionally varied MoS_2_ and CIPS thicknesses using mechanical exfoliation and dry transfer (Figure S26 and S27). These devices span a broad range of channel thicknesses (4–20 nm) and ferroelectric layer thicknesses (75–165 nm), enabling a preliminary and systematic investigation of thickness-dependent performance. Optical microscopy images of the devices, along with the corresponding MoS_2_ and CIPS thicknesses, are presented in Figure S27. As summarized in Table S6, all devices exhibit consistent synaptic behavior, with only modest variations in linearity.


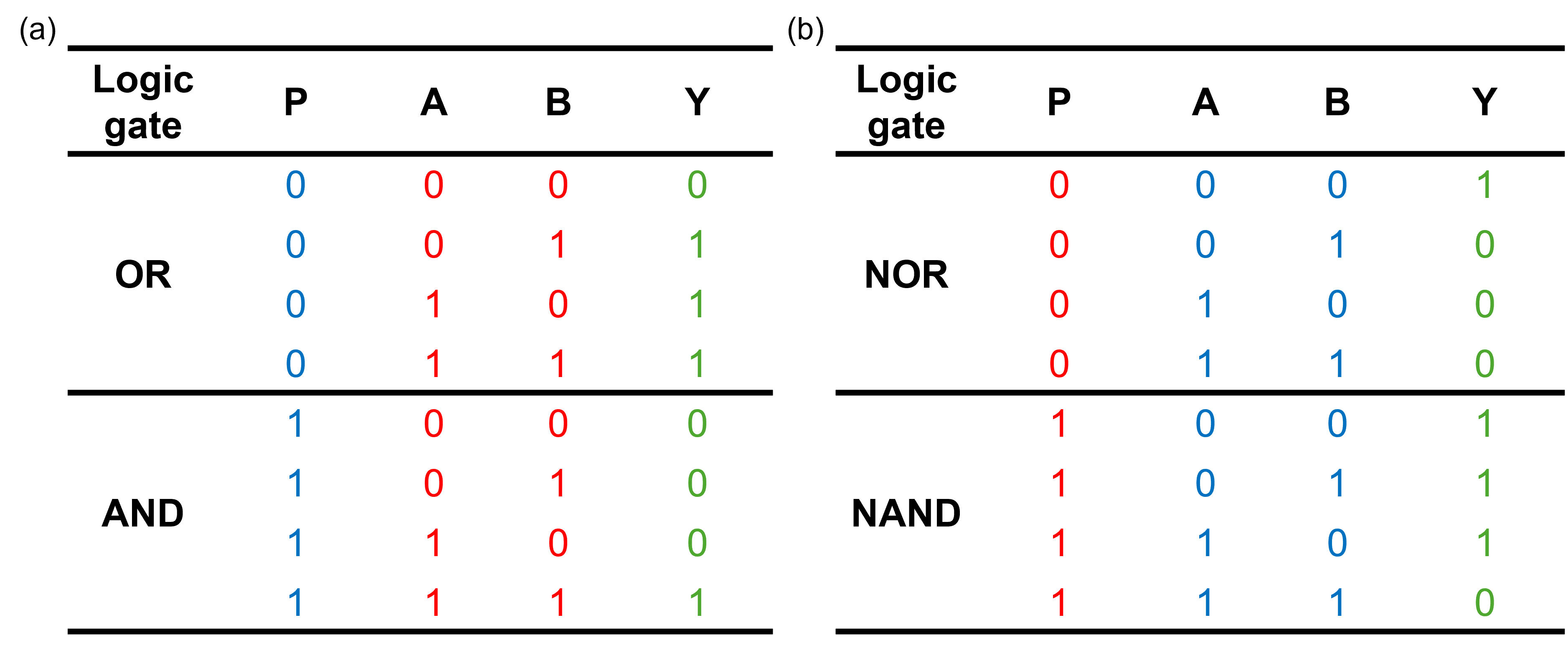


Figure S28. Truth table for the reconfigurable all-optical logic-in-memory operation in the optical-FeFET. (a) Logic operation with 660 nm light as the input signal: one pre-programming signal (P, 450 nm light), two logic inputs (A and B, 660 nm light), and the corresponding output (Y). (b) Logic operation with 450 nm light as the input signal: one pre-programming signal (P, 660 nm light), two logic inputs (A and B, 450 nm light), and the corresponding output (Y).

The truth table is presented in Figure S28, where the absence of a pre-programming signal (“P” in the table) is encoded as “0,” and the presence of a pre-programming signal is encoded as “1”. When 660 nm light is used as the input signal and 450 nm light as the pre-programming signal (Figure S28a), the device performs as an OR gate when P = 0 and as an AND gate when P = 1. Conversely, when 450 nm light is used as the input signal and 660 nm light as the pre-programming signal (Figure S28b), the device functions as a NOR gate when P = 0 and as a NAND gate when P = 1.


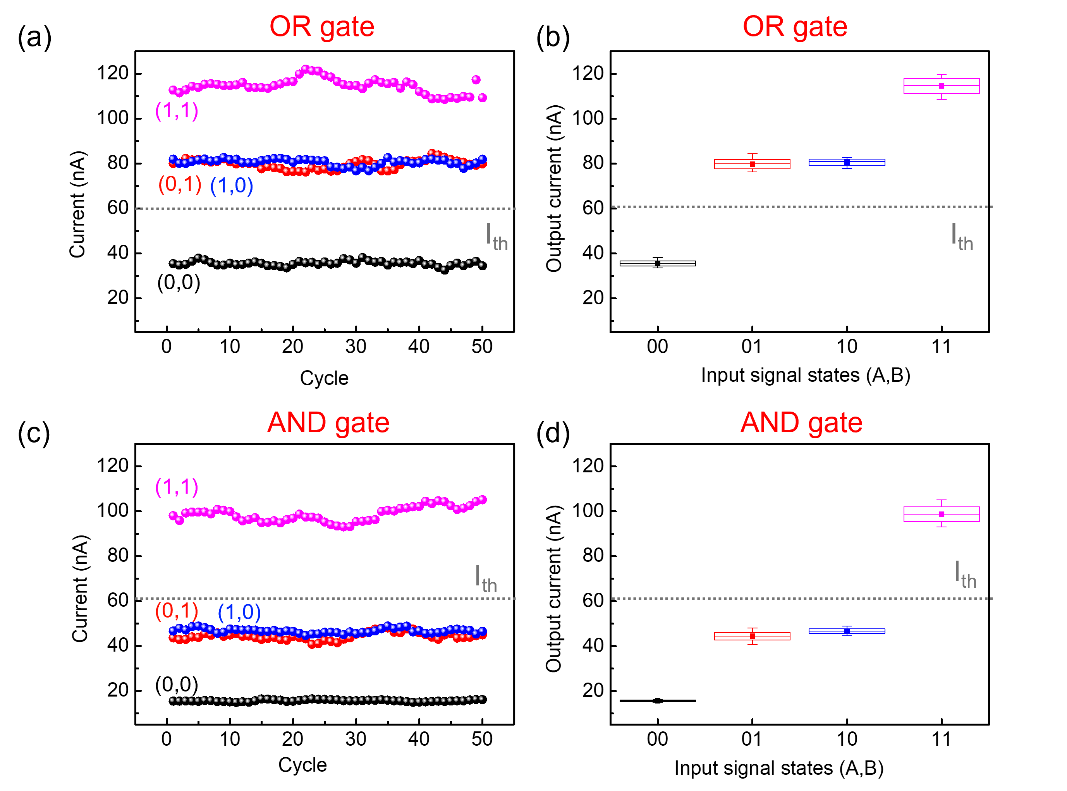


Figure S29. Statistical analysis of Mode 1 logic gates (OR and AND). (a), (c) Output current measured over 50 consecutive operation cycles for different input combinations, (a) OR gate, (b) AND gate. (b), (d) Corresponding box plots of the output current distributions, (b) OR gate, (d) AND gate. A fixed current threshold of 60 nA is used to define logic “0” and logic “1” states, showing separated distributions with an error margin.


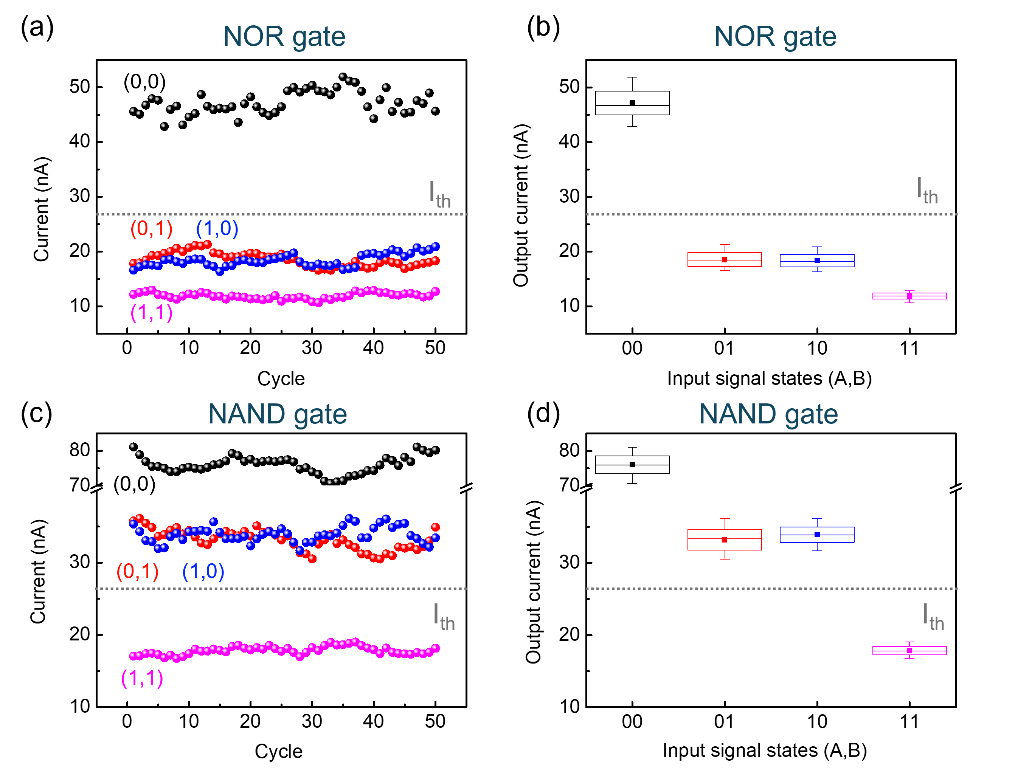


Figure S30. Statistical analysis of Mode 2 logic gates (NOR and NAND). (a), (c) Output current measured over 50 consecutive operation cycles for different input combinations, (a) NOR gate, (b) NAND gate. (b), (d) Corresponding box plots of the output current distributions, (b) NOR gate, (d) NAND gate. A fixed current threshold of 27 nA is used to define logic “0” and logic “1” states, exhibiting separated logic states with an error margin.

Statistical analysis of the output current for the four Boolean logic gates was performed over 50 consecutive operation cycles for each input combination (Figure S29 and S30). Figures S29b and d, and Figures S30b and d present box plots of the output current, showing separated distributions corresponding to logic “0” and logic “1” states.

To define the logic levels, fixed current thresholds were applied: 60 nA for Mode 1 (OR and AND gates in Figure S8) and 27 nA for Mode 2 (NOR and NAND gates in Figure S9). For all four logic gates, the maximum output current of the logic “0” states remain below the threshold, while the minimum output current of the logic “1” states stay above the threshold across all 50 cycles, resulting in an error margin for reliable logic discrimination.

**References**

[1] T. Ahmed, M. Tahir, M. X. Low, Y. Ren, S. A. Tawfik, E. L. H. Mayes, S. Kuriakose, S. Nawaz, M. J. S. Spencer, H. Chen, M. Bhaskaran, S. Sriram, S. Walia, *Adv. Mater.* **2021**, *33*, 2004207.

[2] H. Li, X. Jiang, W. Ye, H. Zhang, L. Zhou, F. Zhang, D. She, Y. Zhou, S. T. Han, *Nano Energy* **2019**, *65*, 104000.

[3] L. Hu, J. Yang, J. Wang, P. Cheng, L. O. Chua, F. Zhuge, *Adv. Funct. Mater.* **2021**, *31*, 5582.

[4] C. M. Yang, T. C. Chen, D. Verma, L. J. Li, B. Liu, W. H. Chang, C. S. Lai, *Adv. Funct. Mater.* **2020**, *30*, 2001598.

[5] Y. X. Hou, Y. Li, Z. C. Zhang, J. Q. Li, D. H. Qi, X. D. Chen, J. J. Wang, B. W. Yao, M. X. Yu, T. B. Lu, J. Zhang, *ACS Nano* **2021**, *15*, 1497.

[6] S. M. Kwon, J. Y. Kwak, S. Song, J. Kim, C. Jo, S. S. Cho, S. J. Nam, J. Kim, G. S. Park, Y. H. Kim, S. K. Park, *Adv. Mater.* **2021**, *33*, 2105017.

[7] X. Shan, C. Zhao, X. Wang, Z. Wang, S. Fu, Y. Lin, T. Zeng, X. Zhao, H. Xu, X. Zhang, Y. Liu, *Adv. Sci.* **2022**, *9*, 2104632.

[8] S. Ge, F. Huang, J. He, Z. Xu, Z. Sun, X. Han, C. Wang, L. B. Huang, C. Pan, *Adv. Opt. Mater.* **2022**, *10*, 200409.

[9] Y. Wang, Y. Zhu, Y. Li, Y. Zhang, D. Yang, X. Pi, *Adv. Funct. Mater.* **2022**, *32*, 2107973.

[10] J. Jiang, W. Xu, Z. Sun, L. Fu, S. Zhang, B. Qin, T. Fan, G. Li, S. Chen, S. Yang, W. Ge, B. Shen, N. Tang, *Small* **2024**, *20*, 2306068.

[11] T. Zhang, C. Fan, L. Hu, F. Zhuge, X. Pan, Z. Ye, *ACS Nano* **2024**, *18*, 16236.

[12] C. Lu, J. Meng, J. Song, T. Wang, H. Zhu, Q. Q. Sun, D. W. Zhang, L. Chen, *Nano Lett.* **2024**, *24*, 1667.

[13] X. Li, Z. Fang, X. Guo, R. Wang, Y. Zhao, W. Zhu, L. Wang, L. Zhang, *ACS Appl. Mater. Interfaces* **2024**, *16*, 27866.

[14] K. Maity, J. F. Dayen, B. Doudin, R. Gumeniuk, B. Kundys, *ACS Appl. Mater. Interfaces* **2023**, *15*, 55948.

[15] J. Jiang, X. Shan, J. Xu, Y. Sun, T. F. Xiang, A. Li, S. ichi Sasaki, H. Tamiaki, Z. Wang, X. F. Wang, *Adv. Funct. Mater.* **2024**, *34*, 2409677.

[16] J. Liang, X. Yu, J. Qiu, M. Wang, C. Cheng, B. Huang, H. Zhang, R. Chen, W. Pei, H. Chen, ACS Appl. Mater. Interfaces 2023, *15*, 9584.

[17] Y. Chen, M. Zhang, D. Li, Y. Tang, H. Ren, J. Li, K. Liang, Y. Wang, L. Wen, W. Li, W. Kong, S. Liu, H. Wang, D. Wang, B. Zhu, *ACS Nano* **2023**, *17*, 12499.

[18] Z. Liu, Y. Wang, Y. Zhang, S. Sun, T. Zhang, Y. J. Zeng, L. Hu, F. Zhuge, B. Lu, X. Pan, Z. Ye, *Adv. Mater.* **2025**, *37*, 2410783.

[19] D. Lembke, A. Kis, *ACS Nano* **2012**, *6*, 10070.

[20] Y. Li, J. Fu, X. Mao, C. Chen, H. Liu, M. Gong, H. Zeng, *Nat. Commun.* **2021**, *12*, 3.

[21] C. Tan, H. Wu, Z. Lin, L. Yang, G. Hu, Z. Wang, *Adv. Funct. Mater.* **2025**, e07832.

[22] D. Qiu, P. Hou, *ACS Appl. Mater. Interfaces* **2023**, *15*, 59671.

[23] Z. Zhang, J. T. Yates, *Chem. Rev.* **2012**, *112*, 5520.

[24] X. Peng, S. Huang, H. Jiang, A. Lu, S. Yu, *IEEE Trans. Comput.-Aided Des. Integr. Circuits Syst.* **2021**, *40*, 2306.

[25] F. Xue, X. He, W. Liu, D.Periyanagounder, C. Zhang, M. Chen, C. H. Lin, L. Luo, E. Yengel, V. Tung, T. D. Anthopoulos, L. J. Li, J. H. He, X. Zhang, Adv. Funct. Mater. 2020, 30, 2004206.

[26] M. Soliman, K. Maity, A. Gloppe, A. Mahmoudi, A. Ouerghi, B. Doudin, B. Kundys, J. F. Dayen, ACS Appl. Mater. Interfaces 2023 15, 15732.

[27] P. Wang, J. Li, W. Xue, W. Ci, F. Jiang, L. Shi, F. Zhou, P. Zhou, X. Xu, Adv. Sci. 2024, 11, 2305679

[28] Z.D. Luo, X. Xia, M. M. Yang, N. R. Wilson, A. Gruverman, ACS Nano 2020, 14, 746.

[29] J. Zha, S. Shi, A. Chaturvedi, H. Huang, P. Yang, Y. Yao, S. Li, Y. Xia, Z. Zhang, W. Wang, H. Wang, S. Wang, Z. Yuan, Z. Yang, Q. He, H. Tai, E. H. T. Teo, H. Yu, J. C. Ho, Z. Wang, Hua Zhang, and C. Tan, Adv. Mater. 2023, 35, 2211598.

[30] N. Savjani, E. A. Lewis, R. A. D. Pattrick, S. J. Haigh, P. O'Brien, RSC Adv. 2014, 4, 35609.

[31] A. C. Gomez, J. Quereda, H. P. Meulen, N. Agraït, G. R. Bollinger, Nanotechnology 2016, 27, 115705

[32] C. H. Chiang, C. C. Lin, Y.C. Lin, C. Y. Huang, C. H. Lin, Y. J. Chen, T. R. Ko, H. L. Wu, W. Y. Tzeng, S. Z. Ho, Y. C. Chen, C. H. Ho, C. J. Yang, Z. W. Cyue, C. L. Dong, C. W. Luo, C. C. Chen, C. W. Chen, Journal of the American Chemical Society 2024, 146, 23278.

[33] S. Fang, Q. Dong, Z. Li, H. Tian, T. Liu, R. Li, X. Jing, L. Yue, C. Li, R. Liu, Q. Li, B. Liu, The Journal of Physical Chemistry C 2023, 127, 8383.

[34] D. Li, X. Hou, F. Kong, K. Wang, X. Hong, ACS Nano 2024, 18, 32890.

[35] P. Li, A. Chaturvedi, H. Zhou, G.Zhang, Q. Li, J. Xue, Z. Zhou, S. Wang, K. Zhou, Y. Weng, F. Zheng, Z. Shi, E. Hang, T. Teo, L. Fang, L. You, Adv. Funct. Mater. 2022, 2201359

[36] R. R. Ma, D. D. Xu, Z. Guan, X. Deng, F. Yue, R. Huang, Y. Chen, N. Zhong, P. H. Xiang, C. G. Duan, Appl. Phys. Lett. 2020, 117, 131102.

[37] X. Q. Yan, X. Zhao, H. Xu, L. Zhang, D. Liu, Y. Zhang, C. Huo, F. Liu, J. Xie, X. Dong, Z. B. Liu, J. G. Tian, J. Mater. Chem. C 2022, 10, 696

[38] C. H. Lee, G. H. Lee, A. M. Zande, W. Chen, Y. Li, M. Han, X. Cui, G. Arefe, C. Nuckolls, T. F. Heinz, J. Guo, J. Hone, P. Kim, Nat. Nanotech. 2014, 9, 676.

[39] F. Ceballos, M. Z. Bellus, H. Y. Chiu, H. Zhao, ACS Nano 2014, 8, 12717.

[40] C. Tan, H. Wu, Z. Lin, L. Yang, G. Hu, Z. Wang, Adv. Funct. Mater. 2026, 36, e07832

[41] D. Qiu, P. Hou, ACS Appl. Mater. Interfaces 2023, 15, 59671.
